# A common outcome set for trials in dementia with Lewy bodies (DLB COS)

**DOI:** 10.1002/trc2.70134

**Published:** 2025-07-11

**Authors:** Joseph P. M. Kane, Rachel L. Fitzpatrick, Sara Betzhold, Gillian Daly, Emily Kalfas, Irina Kinchin, Dag Aarsland, Ken Greaney, Emilia Grycuk, Ann‐Kristin Folkerts, Elke Kalbe, Federico Rodriguez‐Porcel, Ian J. Saldanha, Valerie Smith, John‐Paul Taylor, Rachel Thompson, Kathryn Wyman‐Chick, Iracema Leroi, and The DLB COS Delphi Group

**Affiliations:** ^1^ Centre for Public Health Queen's University Belfast Institute of Clinical Sciences B Royal Victoria Hospital Belfast UK; ^2^ School of Medicine Trinity College Dublin Dublin UK; ^3^ Trinity College Institute of Neuroscience Trinity College Dublin Dublin UK; ^4^ Department of Neuroscience Columbia University New York New York USA; ^5^ Department of Biological and Biomedical Science Trinity College Dublin Dublin UK; ^6^ Centre for Health Policy and Management Trinity College Dublin Dublin UK; ^7^ Department of Psychological Medicine Centre for Healthy Brain Aging Institute of Psychiatry Psychology & Neuroscience King's College London London UK; ^8^ Centre for Age‐Related Diseases Stavanger University Hospital Stavanger Norway; ^9^ Medical Psychology Neuropsychology and Gender Studies Faculty of Medicine and University Hospital Cologne University of Cologne Cologne Germany; ^10^ Medical Faculty and University Hospital University of Cologne Medical Psychology Neuropsychology and Gender Studies Cologne Germany; ^11^ Department of Neurology Medical University of South Carolina Charleston South Carolina USA; ^12^ Department of Epidemiology Center for Clinical Trials and Evidence Synthesis Johns Hopkins Bloomberg School of Public Health Baltimore Maryland USA; ^13^ School of Nursing Midwifery and Health Systems University College Dublin Dublin UK; ^14^ Translational and Clinical Research Institute and NIHR Newcastle Biomedical Research Centre Newcastle University Newcastle upon Tyne UK; ^15^ Dementia UK London UK; ^16^ The Lewy Body Society Wigan UK; ^17^ HealthPartners Struthers Parkinson's Center Minneapolis Minnesota USA; ^18^ Global Brain Health Institute Trinity College Dublin Dublin UK

**Keywords:** Alzheimer's disease and related dementias, consensus, core outcome set, Delphi, dementia, dementia with Lewy bodies, outcome measures, trial methods

## Abstract

**INTRODUCTION:**

Methodological heterogeneity in dementia with Lewy bodies (DLB) trials contributes to publication bias and makes evidence synthesis and meta‐analysis challenging. We aimed to develop a core outcome set for DLB (DLB COS) trials to improve consistency and comparability in DLB research.

**METHODS:**

We conducted a systematic review to identify outcomes and administered a two‐stage Delphi survey to a diverse panel of lay and professional stakeholders. We asked respondents which outcomes should be prioritized and included in DLB COS.

**RESULTS:**

Forty‐nine outcomes were presented to survey respondents. Consensus was reached regarding eight outcomes for the final DLB COS: delusions/paranoia; fluctuations in cognition, attention, and arousal; functioning; global cognition; hallucinations; quality of life; motor parkinsonism; and rapid eye movement sleep behavior disorder.

**DISCUSSION:**

If adopted, DLB COS can enhance the comparability of research findings and facilitate standardization and harmonization.

**Highlights:**

- A systematic review revealed heterogeneity in dementia with Lewy bodies (DLB) study outcomes.
- Our study produced a DLB Core Outcome Set (DLB COS) comprising eight outcomes.
- DLB COS sets the minimum reporting standards for future trials.
- DLB‐specific rating scales incorporating these outcomes are needed.
- Addressing this gap is a strategic priority in DLB research.

## 1 INTRODUCTION

Lewy body dementia (LBD) comprises dementia with Lewy bodies (DLB) and Parkinson's disease (PD) dementia (PDD) and is the second most common form of neurodegenerative dementia.^1^ Although clinically detected in up to 5% of people with dementia,^2^ LBD neuropathology is observed in > 25% in population‐based dementia studies.^3^ DLB is associated with poorer outcomes than in other forms of dementia, such as Alzheimer's disease (AD) dementia, including more frequent hospitalizations^4^ and resource use,^5^ poorer quality of life (QoL), and increased stress in care partners (CPs).^6^

DLB is a clinically heterogeneous disorder. In addition to cognitive and functional impairment, it is characterized by four core symptoms, at least two of which are required for a probable clinical diagnosis: spontaneous parkinsonism; visual hallucinations; rapid eye movement (REM) sleep behavior disorder (RBD); and fluctuations in cognition, attention, and/or alertness.^7^ Heterogeneity in presenting features is common and compounded by pronounced variation in the temporal onset of core features,^8^ and their longitudinal trajectory,^9^ which may reflect variability in co‐pathology.^10^, ^11^ Even patients with severe DLB will not necessarily demonstrate all core features and indicative biomarkers; for example, ≈ 10% of patients with the condition will neither demonstrate parkinsonism nor dopamine transporter deficit on single‐photon emission computed tomography.^12^

Clinical heterogeneity is among the factors challenging measurement of disease severity.^13^ Without a bespoke global DLB outcome measure, trials have adopted a range of global or symptom‐specific alternatives. Global measurements, such as the Clinical Dementia Rating (CDR) scale and Alzheimer's Disease Cooperative Study Clinical Global Impression of Change Scale (ADCS‐CGIC), were designed for use in AD trials and not validated in DLB populations.^13^, ^14^ Available symptom‐specific scales also have limitations; part III of the Movement Disorder Society‐sponsored Unified Parkinson's Disease Rating Scale (MDS‐UPDRS), designed for participants with PD, is useful in detecting motor symptoms in DLB but administration can be complicated by cognitive fluctuations and a lack of precision in early disease.^13^, ^14^

The absence of consensus on DLB outcomes presents significant challenges to the field.^15^ First, the lack of standardization of outcomes, and the adoption of those designed for AD or PD, may impair the detection or interpretation of outcome signals. Second, methodological heterogeneity limits data harmonization, preventing development of larger, more representative sample sizes necessary to provide novel insights into pathogenesis and treatment targets.^16^ Third, heterogeneity arising from variability in outcomes contributes to publication bias and precludes effective evidence synthesis.^17^

A core outcome set (COS) is an agreed standardized set of outcomes that should be measured and reported, as a minimum, in all clinical trials in specific areas of health or health care.^18^, ^19^ The aim of this study was to establish a consensus around a COS for DLB. We did not aim to identify appropriate outcome measurement instruments (OMIs) for these outcomes. Adopting an established methodology, we aimed to engage an international group of multidisciplinary stakeholders through surveys and an online consensus meeting (CM).

## 2 METHODS

### 2.1 Project structure

We adopted the structure and principles established by the Core Outcome Measures in Effectiveness Trials (COMET) initiative, with which the project was registered (https://www.comet‐initiative.org/Studies/Details/2455), and the Consensus‐based Standards for the Selection of Health Measurement Instruments (COSMIN). The protocol was published shortly after data collection commenced.^20^ Ethical approval was granted by the Trinity College Dublin Faculty. Methods and results are reported according to the Core Outcome Set‐Standards for Reporting (COS‐STAR) Statement^21^ (Appendix A in supporting information).

### 2.2 Systematic review

We conducted a PROSPERO‐registered systematic review (SR; CRD42022346808) of outcomes used in DLB studies, comprising three components: quantitative (including pharmacological and non‐pharmacological trials), qualitative, and health economic studies. We searched Central, Medline, Embase, CINAHL, PsycINFO, Web of Science, the National Health Service Economic Evaluation Database (NHS EED), and EconLit on August 16, 2021. The review was conducted using the Covidence platform (Veritas Health Innovation). Inclusion and exclusion criteria are detailed in Table 1 and search terms in Appendix B in supporting information.

**TABLE 1:** Inclusion/exclusion criteria.

	Inclusion criteria	Exclusion criteria
**Article type**	Scientific articles published in peer‐reviewed journals with available full texts	‐ Popular press articles ‐ Editorials, commentaries, etc. ‐ Study protocols ‐ Conference abstracts without full texts ‐ Trial registries
**Study design**	‐ Comparative clinical trials (regardless of randomization) ‐ Pre‐post intervention studies ‐ Case reports of interventions	‐ Observational studies ‐ Systematic reviews and meta‐analyses
**Population/ setting**	‐ People with dementia with Lewy bodies (DLB) ‐ 18 years and older ‐ Both sexes	‐ Parkinson's disease (with and without mild cognitive impairment [PD‐MCI] or Parkinson's disease dementia [PDD]) only ‐ Atypical parkinsonism (e.g., PPS, multisystem atrophy) ‐ Other dementias (e.g., Alzheimer's disease, frontotemporal dementia) ‐ Mixed study samples (e.g., involving participants with different dementia subtypes) without separate reporting for individuals with DLB
**Interventions**	Any pharmacological, surgical, or non‐pharmacological approach for treating motor and non‐motor symptoms in individuals with DLB at any disease stage and in any setting (e.g., outpatients as well as inpatients)	
**Comparators**	‐ Any ‐ None ‐ Placebo/passive control groups/ waitlist ‐ Active control groups (comparison of different pharmacological/ surgical/non‐pharmacological interventions)	
**Outcomes**	Any: ‐ Standardized quantitative assessments, including neurological examinations, assessments, and tests, and questionnaires (self‐andproxy rating) across all domains · Disease severity, motor‐ and non‐motor symptoms · Patient‐reported outcome measures (PROMs) including quality of life · Biomarkers, imaging outcomes · Changes in housing and care situation (e.g., institution) · Caregiver outcomes included in the patient‐targeted interventions ‐ Qualitative approaches to evaluate intervention success across all domains (e.g., outcomes obtained through patient interviews, focus groups) ‐ Economic evaluation outcomes (cost analysis, cost‐effectiveness analysis, cost‐utility analysis, cost‐benefit analysis)	

We included studies of adult participants with DLB at any disease stage and in any setting, with no restriction on age, sex, or gender. We excluded studies that reported all‐cause dementia groups without separately providing DLB results. Studies involving cohorts with LBD, comprising DLB and PDD, were included, but those that included PDD cohorts alone were excluded. Two of three co‐authors (A.‐K.F., I.K., S.B.) independently reviewed each abstract and conducted full‐text screening and data extraction. Disagreements were resolved through discussion among the three co‐authors.

Extracted outcomes were arranged into an outcome matrix^19^ and iteratively organized into categories for the purposes of presenting numerous preliminary outcomes to participants in a logical and coherent manner. This COS focused on clinical and economic outcomes; because access to DLB biomarkers is variable, even in high‐income countries, biomarker outcomes were not extracted.

**RESEARCH IN CONTEXT**

1. **Systematic Review**: A systematic review comprising three components (quantitative studies, qualitative studies, and health economic studies) demonstrated heterogeneity in outcomes in dementia with Lewy bodies (DLB) studies. This heterogeneity inhibits data synthesis and harmonization of DLB data.
2. **Interpretation**: An iterative Delphi process, involving people with DLB and their care partners, as well as a geographically and disciplinarily diverse group of professional stakeholders, reached a consensus on a core outcome set for DLB (DLB COS) consisting of eight outcomes (delusions/paranoia; fluctuations in cognition, attention, and arousal; functioning; global cognition; hallucinations; motor parkinsonism; quality of life; and rapid eye movement sleep behavior disorder).
3. **Future Directions**: The DLB COS represents the minimum reporting requirements for future DLB trials. These outcomes should also be considered for integration into DLB‐specific rating scales, the absence of which represent an unmet strategic need in the field.

### 2.3 Delphi survey

A Delphi process helped determine which outcomes were most relevant to a multidisciplinary range of stakeholders. A Delphi process is a research method that gathers expert opinions through multiple rounds of surveys, adopting an iterative approach that lends itself to complex issues, such as DLB outcomes, in which no consensus otherwise exists.^22^

Clinical and research experts in DLB were identified through their community profile and publications.^23^ Geriatric psychiatrists, neurologists, and geriatricians were represented, as well as experts outside of these specialties to reflect the multidisciplinary nature of DLB research and care. Acknowledging that the DLB research is largely confined to high‐income countries,^16^ clinicians and researchers from low‐ and middle‐income countries (LMICs) engaged in a global brain program were approached. J.K. presented the study protocol to the International LBD Conference on June 16, 2022; attendees were invited to join the survey. By e‐mail, prospective respondents were linked to Participant Information Sheet, electronic consent form, and the Round 1 (R1) survey. Professional respondents (PRs) are listed in Appendix C in supporting information.

People with DLB and their CPs (“lay respondents,” LRs) were recruited via an e‐mail circulated by advocacy organizations (Lewy Body Society, Lewy Body Dementia Association, Parkinson's UK, and Parkinson's Association of Ireland). The same hyperlink provided to PRs was provided to prospective LRs in this e‐mail. The only inclusion criterion for participation was that respondents were either people with DLB, or a CP for a person with DLB now or in the past. All participants provided consent via electronic form. Although no language requirements were specified, all study materials were available in English only.

#### 2.3.1 Delphi R1

R1 was administered online using DelphiManager. Using a nine‐point Likert scale, respondents rated the importance of inclusion of each outcome in DLB COS. Additionally, respondents were invited to suggest modifications to the listed outcomes or propose any outcomes not already included. Before launch, all surveys were piloted among professional and lay stakeholders. Detailed descriptions in accessible language were provided for each outcome in R1 and Round 2 (R2) and referred to at the CM.

In keeping with the Grading of Recommendations Assessment, Development, and Evaluation (GRADE) framework^24^ and the Research and Development (RAND) appropriateness method^25^ an outcome passed to the next stage when ≥ 70% of respondents rated inclusion of an outcome as 7 to 9 (“critical for inclusion”) and ≤ 15% of respondents rated the outcome as 1 to 3 (“not important”).

#### 2.3.2 Delphi R2

Before R2, the study steering committee (S.C., D.A., E.K., J.P.M.K., A.‐K.F., I.K., I.L., F.R.‐P., I.J.S., V.S., J.‐P.T., R.T.) reviewed responses to R1, including suggestions submitted through the “free text” function. Consequently, the study team altered the format of the R2 survey, and changed one outcome (“sleep”), as discussed in the Results section. All LRs and PRs were invited to participate in R2, administered via Qualtrics. Each participant ranked what they considered to be the 10 outcomes most critical for inclusion. Data on whether respondents were PRs or LRs was not collected during R2.

### 2.4 Consensus meeting

All R1 and R2 respondents were invited to an online CM at which outcomes rated critical for inclusion by ≥ 70%, and not important by ≤ 15% of R1 respondents, were presented. R2 data were presented as the proportion of respondents who nominated each of the remaining outcomes as among the 10 most important for inclusion. When an R2 outcome was rated among the 10 most important for inclusion by ≥ 70% of responders, it was included in DLB COS when ≥ 70% of attendees, including at least one LR, voted to ratify its inclusion.

We recognized that 6 to 10 outcomes are recommended for a COS,^26^ and that adoption of some flexibility on these thresholds was required. It was agreed before the meeting if fewer than six outcomes were identified through the method above, that those included in < 70% of R2 responses would still be discussed and voted into the DLB COS if approved by ≥ 70% of attendees. When two or more similar outcomes could be combined, attendees would be invited to reformulate outcomes, which could be included in the DLB COS with the approval of ≥ 70% of attendees.

### 2.5 OMIs

COMET and COSMIN recommend that after the selection of outcomes, suitable OMIs are identified, using existing SRs, and other optional sources.^27^ Two literature reviews investigating OMIs in DLB^13^, ^15^ and a SR of economic outcomes in DLB^5^ were published shortly before this project began, highlighting the poor suitability of OMIs often used in DLB trials. Notably, these papers cited insufficient data to recommend specific OMIs.^15^ Similarly, we did not aim to identify OMIs for outcomes.

## 3 RESULTS

### 3.1 Systematic reviews

**FIGURE 1:**
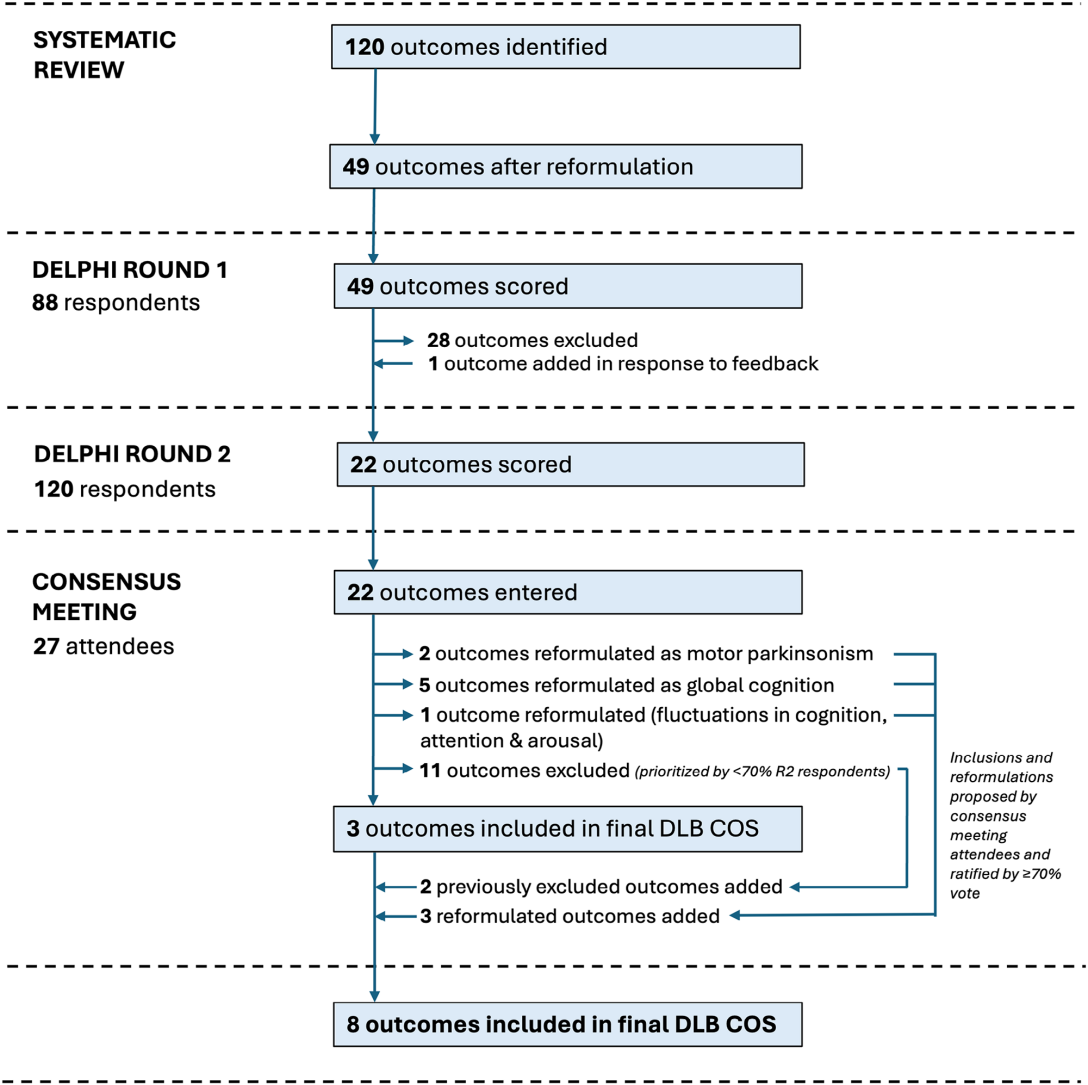
Summary flowchart of outcomes included, excluded, and reformulated throughout the DLB COS development process. DLB COS, dementia with Lewy bodies common outcome set; R2, round 2.

Our SR of quantitative, qualitative, and health economics studies yielded 2508 records, of which 251 underwent full‐text review (Appendix D in supporting information). From 127 included studies, 120 individual outcomes were identified. As we wished to mitigate responder fatigue and dropout, 120 outcomes were reformulated to 49 for the R1 survey (Figure 1) and categorized into five categories (Table 2).

**TABLE 2:** Outcomes identified through systematic review.

Cognitive ability & fluctuations (*n* = 8)	
Attentional deficits/psychomotor slowing	Language/communication abilities
Executive functions (including working memory)	Memory
Fluctuations in cognition	Social cognition
Global cognition	Visuocognition

**Functioning & quality of life (** *n* ** = 6)**	
Activities of daily living	Care partner quality of life
Care partner burden & distress	Global clinical impression
Care partner mood/depressive symptoms	Quality of life

**Health economic outcomes (** *n* ** = 7)**	
Cost of home improvements	Medical costs paid out of pocket
Costs of lost productivity	Transportation costs
Cost of social services	Unpaid caregiver costs
Medical costs paid by the state/medical insurance	

**Psychiatric & sleep‐related outcomes (** *n* ** = 13)**	
Aggression	Euphoria
Agitation	Hallucinations
Anxiety/phobias	Irritability/lability
Apathy Delusions/paranoia	Non–sleep‐related nighttime behavior changes/day–night reversal
Depression/dysphoria	Sleep
Disinhibition	Undirected movement/restlessness

**Motor & non‐motor Parkinsonism (** *n* **= 15)**	
Appetite and eating abnormalities	Problems with thermoregulation
Akathisia	Problems with urinary functioning
Constipation/diarrhea	Salivating/drooling/sialorrhea
Falling	Sexual dysfunction
Gait/walking	Speech
On versus off states	Strength
Parkinsonism	Swallow
Problems with cardiovascular functioning	

### 3.2 Delphi survey

#### 3.2.1 R1

We identified 117 professional experts. Two opted out of the study, and 48 PRs (41%) working in 12 countries completed the R1 survey. PRs comprised neurologists (*n* = 19), geriatric psychiatrists (*n* = 10), specialist nurses (*n* = 5), geriatric physicians (*n* = 3), researchers (*n* = 3), health economists (*n* = 2), clinical psychologists (*n* = 2), a care home nursing director (*n* = 1), a speech and language therapist (*n* = 1), an occupational therapist (*n* = 1), and a physiotherapist (*n* = 1). LRs comprised 40 individuals from four countries.

**FIGURE 2:**
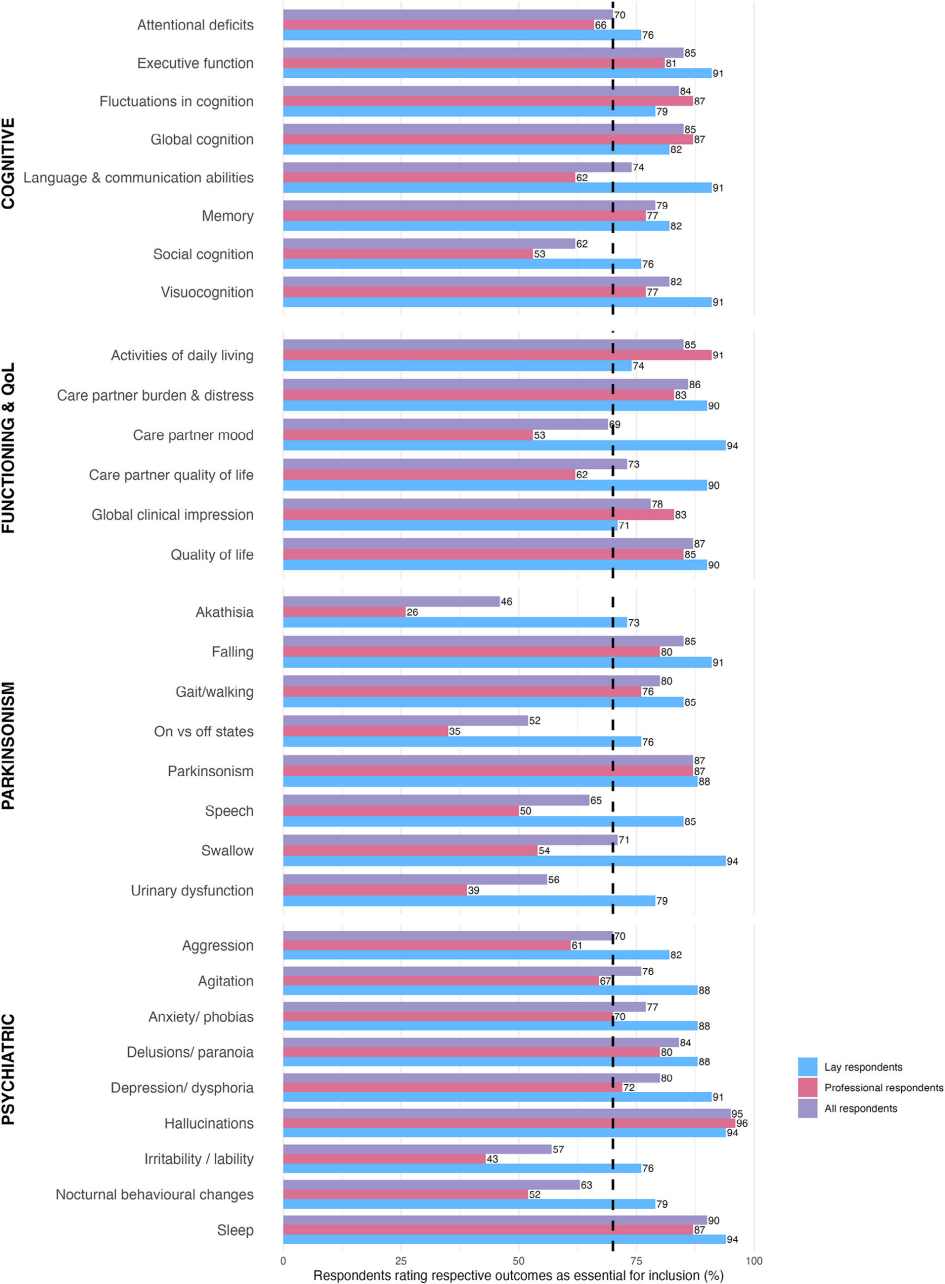
Outcomes rated as “essential for inclusion” (scored 7–9) by Round 1 respondents (*n* = 88; *n* = 48 professional respondents, *n* = 40 lay respondents).

PRs supported inclusion of 16 outcomes (Figure 2, Appendix E in supporting information) and the exclusion of 7. LRs supported the inclusion of 31 outcomes and did not exclude any. The combined group supported inclusion of 22 outcomes and the exclusion of 1.

#### 3.2.2 R2

**FIGURE 3:**
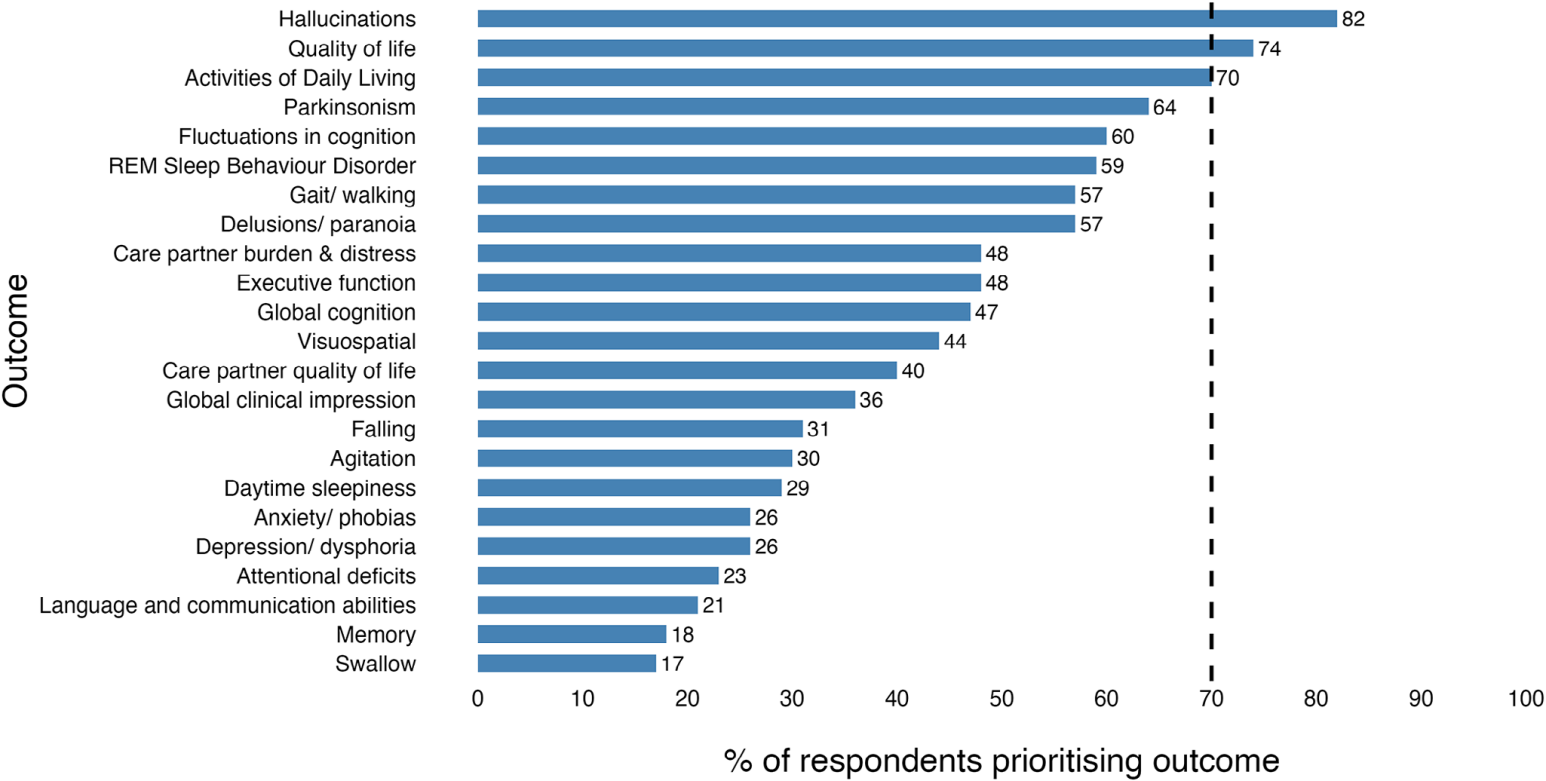
Outcomes prioritized by Round 2 respondents (*n* = 120).

R1 PRs reported that the “sleep” outcome was too broad, and that it should be split into “REM sleep behavior disorder (RBD)” and “daytime sleepiness.” When these two outcomes were added to the other 21 supported by R1 respondents, 23 outcomes were included in the R2 survey, which 120 respondents completed (Figure 1). Three outcomes—hallucinations, QoL, and activities of daily living (ADLs)—were included in ≥ 70% of respondents’ 10 preferred outcomes (Figure 3). Data were not collected in relation to respondents’ status as PRs or LRs.

### 3.3 Consensus meeting

An online CM was held on November 7, 2023 and attended by 27 respondents, of whom 8 (30%) were LRs. After R1 and R2 results were presented, an open discussion was facilitated by J.K. Throughout the meeting attendees were encouraged to share their ideas and proposals for DLB COS, on which the rest of the group was invited to vote. Attendees were also reminded that the discussion should relate to outcomes, not OMIs. A consensus was reached that hallucinations, ADLs, and QoL should be included. Although it was proposed that “hallucinations” would be changed to “visual hallucinations,” this was not met with a consensus; however, there was support for replacing “(ADLs)” with “functioning.” Attendees understood that 6 to 10 outcomes are recommended by COSMIN and were invited to propose and discuss, based on the survey results presented, whether additional or reformulated outcomes should be added to the three outcomes initially included. It was proposed that five outcomes included by > 60% but < 70% of R2 participants (motor parkinsonism; RBD; fluctuations in cognition; delusions/paranoia; and difficulties with gait, walking, steadiness, or balance; Figures 1 and 3) should be included, and this was supported by ≥ 70% of CM attendees. It was subsequently agreed that “fluctuations in cognition” should become “fluctuations in cognition, attention, and arousal” and that difficulties with gait, walking, steadiness, or balance should be included within the “motor parkinsonism” outcome. Finally, cognitive outcomes were distributed across several subcategories: global cognition was included in 50% of R2 respondents’ top 10 ranked outcomes, but executive function (52%), visuocognition (45%), attention (25%), and memory (22%) were also ranked highly. A consensus was reached that global cognition should be included in DLB COS (Figure 3). All other outcomes, all included in < 50% of R2 respondents’ choices, were not put to a vote at the CM.

## 4 DISCUSSION

**FIGURE 4:**
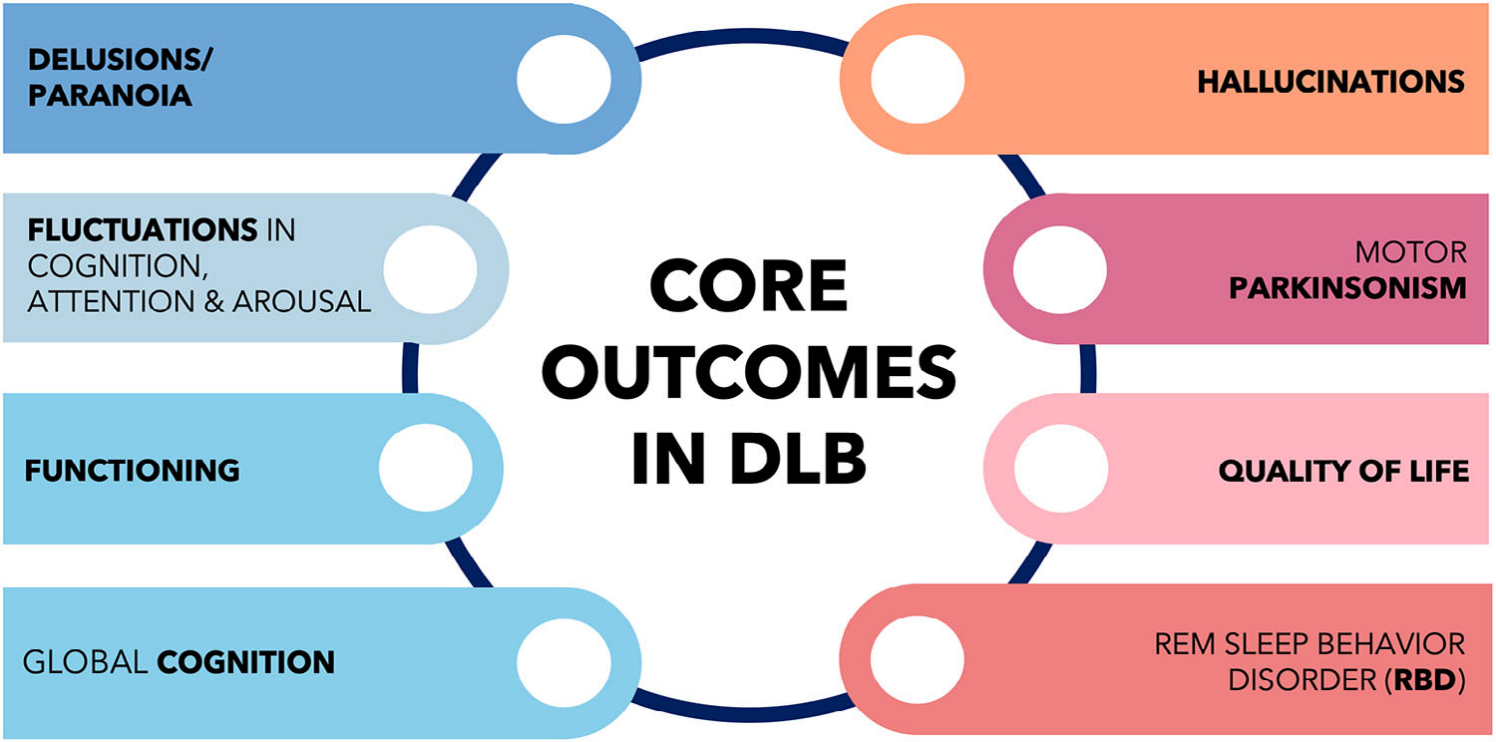
Final core outcome set for DLB (DLB COS). DLB COS, dementia with Lewy bodies common outcome set; REM, rapid eye movement.

Using an established and systematic study design,^19^, ^20^ we have developed international consensus regarding a DLB COS comprising eight outcomes that we propose should represent the minimum reporting requirements for clinical trials in DLB (Figure 4).

Central to the study's methodology was consultation with people with DLB and their CPs.^19^ Underrepresentation of the perspectives of patients and CPs in outcome identification is common in health‐care research,^28^, ^29^ and inadequate stakeholder involvement has been identified as a significant barrier to COS adoption.^26^, ^30^ Insufficient lay stakeholder engagement is particularly acute in DLB research.^31^ Although two recent studies have engaged people with DLB and their CPs on prioritization of research themes,^32^, ^33^ neither specifically addressed outcomes in trial design. Several symptoms ranked highly by R2 respondents, including cognitive impairment, fluctuations, and Parkinsonism, mirror those identified as “most bothersome” in a survey of people with LBD and their CPs.^33^ However, a strength of the COS methodology is the ability to look beyond symptoms; two of the first three outcomes included in DLB COS related broader constructs (QoL and functioning).

Inclusion of both LRs and PRs required reconciliation of their respective perspectives; LRs supported the inclusion of 31/49 (63%) of outcomes and excluded none, while PRs reached consensus on 16 outcomes, several of which were included in the final COS. PRs may have placed greater emphasis on feasibility of DLB COS than inclusivity of symptoms, while the LRs may have prioritized the burden of individual symptoms, rather than the importance that each outcome is measured in clinical trials. A greater understanding of these perspectives might have been achieved by qualitative analysis, but any differences between LRs and PRs only support the importance of engaging multiple stakeholders in COS projects. As demonstrated by the ranking exercise we used for R2, these differences are not irreconcilable, and the list of outcomes discussed in the CM, and those included in DLB COS, were acceptable to both groups.

Another critical component of this study's design was the established methodology used,^18^, ^19^ which has permitted COSs to optimize adoption and impact. A rheumatoid arthritis COS was fully adopted in 82% of trials,^34^ and endorsed by both the US Food and Drug Administration and the European Medicines Agency.^35^ Recent guidance from the World Health Organization states that COSs “should be considered for all trials, to enable the results of studies being compared, contrasted and combined (for example, in later meta‐analyses) as appropriate.”^36^ Funders such as the UK National Institute for Health Research has recommended that COSs are considered in grant proposals where applicable; 38% of applicants have searched for COSs in the development of their submissions since.^37^

The outcomes comprising DLB COS broadly demonstrate content validity. Inclusion of cognition and functioning was anticipated as impairment in both are essential in diagnosis of the clinical dementia syndrome.^38^ Similarly, four of the outcomes identified—hallucinations; motor parkinsonism; RBD; and fluctuations in cognition, attention, and arousal—align with core features of DLB.^7^ As a supportive clinical feature, carrying less diagnostic weight than the core symptoms, the inclusion of delusions may reflect its significant clinical impact in DLB, particularly on hospitalization^4^ and CP stress.^6^ QoL is a concept well recognized by both LRs and PRs and may represent a more acceptable multidimensional outcome than the clinical global impression adopted as primary outcome in some DLB trials.^14^, ^39^ QoL has been a secondary outcome in recent landmark AD trials^40^, ^41^ but no DLB trials currently listed on ClinicalTrials.gov include QoL (Appendix F in supporting information).

We did not identify OMIs corresponding to the outcomes identified. This is not recommended by COSMIN when addressed by recent high‐quality reviews;^19^ significant limitations of the OMIs available are addressed in greater detail in recent publications.^13^, ^15^ Like our SR, these describe the absence of a DLB‐specific OMI and the reliance on those designed for use in AD and PD. The Mini‐Mental State Examination (MMSE) and Montreal Cognitive Assessment (MoCA), while widely adopted, demonstrate limited sensitivity in DLB,^13^ not least due to their focus on amnesia, rather than deficits in cognitive domains characteristically impaired in DLB.^42^ Beyond choice of OMI, measurement of cognition in DLB is also challenged by fluctuations, and the secondary effects of RBD and motor parkinsonism.^42^ The Neuropsychiatric Inventory (NPI)^43^ provides a multidimensional measurement of hallucinations and delusions but lacks specificity and granular detail on the sensory modality of hallucinations. MDS‐UPDRS,^44^ although frequently used to evaluate parkinsonism, can be burdensome to the patient, and its composite score can mean that improvements in one feature may mask deteriorations in others.^13^ It may also lack precision in early disease.^45^ Alzheimer's Disease Cooperative Study Activities of Daily Living and Quality of Life in Alzheimer's Disease are measurements of functioning and QoL, two of the outcomes most highly prioritized in R2; however, neither have been validated in DLB.^13^ The Mayo Sleep Questionnaire^46^ and the Clinical Assessment of Fluctuations scale^47^ have been validated in DLB, but the latter demonstrates limited interrater reliability, and the former requires a bed partner informant, which often is not available.

Our abstention in identifying appropriate OMIs is not unusual; 65% of COSs do not make such recommendations.^48^ This provides prospective adopters greater flexibility than would be provided by a more proscriptive approach that included recommended OMIs. Although not immediately ameliorating harmonization or heterogeneity among DLB datasets, DLB COS adoption would partially address the previously unmet need of an absence of patient‐ and CP‐informed outcomes in trials and mitigate publication bias. While not providing complete standardization, DLB COS offers a template for trial standardization that does not otherwise currently exist. These merits notwithstanding, there remains an immediate unmet need for multi‐stakeholder, COSMIN‐informed appraisal and agreement on OMIs despite their clear limitations discussed above.

The strengths of this project include our rigorous application of COSMIN and COMET guidelines, and engagement of a diverse group of professional and lay stakeholders. It is the only project to have engaged LRs specifically on the topic of research outcomes. Our adoption of a broad SR reduced the risk of excluding important outcomes, further decreased by providing respondents with three opportunities to propose alternative outcomes.

The project is limited by being conducted exclusively in English, potentially excluding participants from non‐anglophone countries and LMICs. By recruiting LRs through charity organizations and in delivering the survey online, we did not access marginalized, isolated, or sensory impaired communities. Due to an error in our survey design, we were unable to determine whether respondents to R2 were LRs or PRs, but this is unlikely to differ significantly from the proportions observed in R1 as the total number of respondents was similar in R1 (*n* = 98) and R2 (*n* = 120). Another limitation was our exclusion of observational studies from our SR. Because the aims of the project related primarily to trial design, our searches focused on trials, but a broader search that included clinical or research priorities of people with DLB, or studies exploring novel OMIs,^49^ might have informed inclusion of alternative domains. Finally, compared to the number of respondents to R1 and R2, our CM was attended by a smaller group of participants (*n* = 27). Given the reorganization of some outcomes and the adoption of five outcomes with less that the a priori threshold for consensus, it could be argued that these attendees’ opinions carried more weight than those unable to attend. Neither COMET nor COSMIN recommend a minimum proportion of respondents required, prioritizing engagement of relevant stakeholders over the quantity of attendees. We consider the balance between LRs and PRs at our CM sufficiently represented the broader group, and the face validity of DLB COS suggests that the consensus at the CM was not incongruent with that of the wider group.

Further directions for DLB COS include its dissemination and encouragement of researchers, funders, and regulatory agencies to adopt it. Prospective adopters should recognize that DLB COS represents a minimum reporting requirement alone, and that it would neither preclude investigation of additional outcomes like biomarkers, nor analysis of subcomponents of an included outcome (such as visuospatial ability as part of global cognition). Periodic review of registered and published trials will be conducted to determine uptake,^13^, ^15^ and development of any DLB‐specific rating scale should consider use of DLB COS in psychometric evaluation.

To conclude, DLB COS has been established by consensus and should represent the minimum reporting requirements in DLB trials. Widespread adoption could reduce methodological heterogeneity, support evidence synthesis and harmonization, and influence clinical and research policy.

## AUTHOR CONTRIBUTIONS

Iracema Leroi secured the funding for this project. The steering committee, comprising Dag Aarsland, Elke Kalbe, Joseph P. M. Kane, Ann‐Kristin Folkerts, Irina Kinchin, Iracema Leroi, Federico Rodriguez‐Porcel, Ian J. Saldanha, Valerie Smith, John‐Paul Taylor, and Rachel Thompson, defined the overall aims and scope of the consensus. Sara Betzhold, Emily Kalfas, and Emilia Grycuk led protocol development in collaboration with the steering committee. Ann‐Kristin Folkerts led systematic review in collaboration with the steering committee. Gillian Daly and Rachel Fitzpatrick led survey development, data collection, and analysis in collaboration with steering committee. Rachel Fitzpatrick and Joseph P. M. Kane drafted the manuscript, and all authors critically reviewed the manuscript drafts.

## CONFLICT OF INTEREST STATEMENT

D.A. has received research support and/or honoraria from Astra‐Zeneca, H. Lundbeck, Novartis Pharmaceuticals, Evonik, Roche Diagnostics, GE Health, and Sanofi, and served as paid consultant for H. Lundbeck, Eisai, Heptares, Eli Lilly, Enterin, Acadia, EIP Pharma, Biogen, and Takeda Pharmaceuticals. J.K. has been awarded research grants from Alzheimer's Research UK, the Lewy Body Society, and the Irish Health Research Board (HRB) and has received consultation fees from Takeda Pharmaceuticals and honoraria from Eisai Co. Ltd. J.K. and I.L. are directors of Lewy Body Ireland. I.L. has been awarded research grants from Horizon 2020, the EU Joint Programme–Neurodegenerative Disease Research (JPND), Erasmus Plus, HRB, the Irish Research Council, and the UK National Institute for Health Research. F.R.P. is funded by the National Institutes of Health (NIH) and also receives royalties from Cambridge University Press. J.‐P.T. has acted as chief investigator for UK component of a Phase II trial in DLB (EIP Pharma). He has consulted for Heptares‐Sosei and Kwayo‐Kirin and received speaker fees from GE Healthcare and Bial. S.B., G.D., A.F., R.F., E.G., K.G., E.K., I.K., I.S., V.S., R.T., and K.W.C. have no interests to declare. Author disclosures are available in the supporting information.

## CONSENT STATEMENT

All participants in this study provided informed consent.

## Supporting information



Supporting Information

Supporting Information

Supporting Information

Supporting Information

Supporting Information

Supporting Information

Supporting Information
