# Supplementary material for: A common outcome set for trials in dementia with Lewy bodies (DLB COS)
Source: Alzheimers Dement (N Y). 2025 Jul 11;11(3):e70134. doi: 10.1002/trc2.70134 (PMC12254044; doi:10.1002/trc2.70134)
Supplement: Supplementary file 1 — Supporting Information [file TRC2-11-e70134-s006.pdf]

ICMJE DISCLOSURE FORM

**Date:**	5/30/2025
**Your Name:**	Joseph Kane
**Manuscript Title:**	A common outcome set for trials in dementia with Lewy bodies (DLB COS)
**Manuscript Number (if known):**	TRCI-D-25-00068R1

In the interest of transparency, we ask you to disclose all relationships/activities/interests listed below that are related to the content of your manuscript. "Related" means any relation with for-profit or not-for-profit third parties whose interests may be affected by the content of the manuscript. Disclosure represents a commitment to transparency and does not necessarily indicate a bias. If you are in doubt about whether to list a relationship/activity/interest, it is preferable that you do so.

The author's relationships/activities/interests should be defined broadly. For example, if your manuscript pertains to the epidemiology of hypertension, you should declare all relationships with manufacturers of antihypertensive medication, even if that medication is not mentioned in the manuscript.

In item #1 below, report all support for the work reported in this manuscript without time limit. For all other items, the time frame for disclosure is the past 36 months.

		Name all entities with whom you have this relationship or indicate none (add rows as needed)	Specifications/Comments (e.g., if payments were made to you or to your institution)
**Time frame: Since the initial planning of the work**			
1	All support for the present manuscript (e.g., funding, provision of study materials, medical writing, article processing charges, etc.) **No time limit for this item.**	☒ None	
			Click the tab key to add additional rows.
**Time frame: past 36 months**			
**2**	Grants or contracts from any entity (if not indicated in item #1 above).	☐ **None**	
		Lewy Body Society Project Grant	
**3**	Royalties or licenses	☒ **None**	

		Name all entities with whom you have this relationship or indicate none (add rows as needed)	Specifications/Comments (e.g., if payments were made to you or to your institution)
**4**	Consulting fees	☒ **None**	
**5**	Payment or honoraria for lectures, presentations, speakers bureaus, manuscript writing or educational events	☐ **None**	
		Lewy Body Academy	Paid to me
**6**	Payment for expert testimony	☒ **None**	
**7**	Support for attending meetings and/or travel	☐ **None**	
		Alzheimer's Research UK travel grant	Paid to my institution – contribution towards travel to ILBDC to present this work
**8**	Patents planned, issued or pending	☒ **None**	
**9**	Participation on a Data Safety Monitoring Board or Advisory Board	☐ **None**	
		Lewy Body Society	Unpaid member of Scientific Advisory Committee
**10**	Leadership or fiduciary role in other board, society, committee or advocacy group, paid or unpaid	☐ **None**	
		Lewy Body Ireland	Unpaid board member and secretary

		Name all entities with whom you have this relationship or indicate none (add rows as needed)	Specifications/Comments (e.g., if payments were made to you or to your institution)
**11**	Stock or stock options	☒ **None**	
**12**	Receipt of equipment, materials, drugs, medical writing, gifts or other services	☒ **None**	
**13**	Other financial or non-financial interests	☒ **None**	

Please place an "X" next to the following statement to indicate your agreement:

☒ I certify that I have answered every question and have not altered the wording of any of the questions on this form.

ICMJE DISCLOSURE FORM

Date: 6/3/2025

Your Name: Rachel L. Fitzpatrick

Manuscript Title: A common outcome set for trials in dementia with Lewy bodies (DLB COS)

Manuscript Number (if known): TRCI-D-25-00068R1

In the interest of transparency, we ask you to disclose all relationships/activities/interests listed below that are related to the content of your manuscript. "Related" means any relation with for-profit or not-for-profit third parties whose interests may be affected by the content of the manuscript. Disclosure represents a commitment to transparency and does not necessarily indicate a bias. If you are in doubt about whether to list a relationship/activity/interest, it is preferable that you do so.

The author's relationships/activities/interests should be defined broadly. For example, if your manuscript pertains to the epidemiology of hypertension, you should declare all relationships with manufacturers of antihypertensive medication, even if that medication is not mentioned in the manuscript.

In item #1 below, report all support for the work reported in this manuscript without time limit. For all other items, the time frame for disclosure is the past 36 months.

		Name all entities with whom you have this relationship or indicate none (add rows as needed)	Specifications/Comments (e.g., if payments were made to you or to your institution)
Time frame: Since the initial planning of the work			
1	All support for the present manuscript (e.g., funding, provision of study materials, medical writing, article processing charges, etc.) **No time limit for this item.**	☒ None	
			Click the tab key to add additional rows.
Time frame: past 36 months			
**2**	Grants or contracts from any entity (if not indicated in item #1 above).	☒ **None**	
**3**	Royalties or licenses	☒ **None**	

		Name all entities with whom you have this relationship or indicate none (add rows as needed)	Specifications/Comments (e.g., if payments were made to you or to your institution)
**4**	Consulting fees	☒ **None**	
**5**	Payment or honoraria for lectures, presentations, speakers bureaus, manuscript writing or educational events	☒ **None**	
**6**	Payment for expert testimony	☒ **None**	
**7**	Support for attending meetings and/or travel	☒ **None**	
**8**	Patents planned, issued or pending	☒ **None**	
**9**	Participation on a Data Safety Monitoring Board or Advisory Board	☒ **None**	
**10**	Leadership or fiduciary role in other board, society, committee or advocacy group, paid or unpaid	☒ **None**	

		Name all entities with whom you have this relationship or indicate none (add rows as needed)	Specifications/Comments (e.g., if payments were made to you or to your institution)
**11**	Stock or stock options	☒ **None**	
**12**	Receipt of equipment, materials, drugs, medical writing, gifts or other services	☒ **None**	
**13**	Other financial or non-financial interests	☒ **None**	

Please place an "X" next to the following statement to indicate your agreement:

☒ I certify that I have answered every question and have not altered the wording of any of the questions on this form.

ICMJE DISCLOSURE FORM

**Date:**	6/16/2025
**Your Name:**	Sara Betzhold
**Manuscript Title:**	A common outcome set for trials in dementia with Lewy bodies (DLB COS)
**Manuscript Number (if known):**	TRCI-D-25-00068R1

In the interest of transparency, we ask you to disclose all relationships/activities/interests listed below that are related to the content of your manuscript. "Related" means any relation with for-profit or not-for-profit third parties whose interests may be affected by the content of the manuscript. Disclosure represents a commitment to transparency and does not necessarily indicate a bias. If you are in doubt about whether to list a relationship/activity/interest, it is preferable that you do so.

The author's relationships/activities/interests should be defined broadly. For example, if your manuscript pertains to the epidemiology of hypertension, you should declare all relationships with manufacturers of antihypertensive medication, even if that medication is not mentioned in the manuscript.

In item #1 below, report all support for the work reported in this manuscript without time limit. For all other items, the time frame for disclosure is the past 36 months.

		**Name all entities with whom you have this relationship or indicate none (add rows as needed)**	**Specifications/Comments (e.g., if payments were made to you or to your institution)**
		**Time frame: Since the initial planning of the work**	
1	All support for the present manuscript (e.g., funding, provision of study materials, medical writing, article processing charges, etc.) **No time limit for this item.**	☐ None	
		HRB Summer Research Scholar Recipient in 2021	2400 Euros
			Click the tab key to add additional rows.
		**Time frame: past 36 months**	
**2**	Grants or contracts from any entity (if not indicated in item #1 above).	☒ **None**	
**3**	Royalties or licenses	☒ **None**	

		Name all entities with whom you have this relationship or indicate none (add rows as needed)	Specifications/Comments (e.g., if payments were made to you or to your institution)
**4**	Consulting fees	☒ **None**	
**5**	Payment or honoraria for lectures, presentations, speakers bureaus, manuscript writing or educational events	☒ **None**	
**6**	Payment for expert testimony	☒ **None**	
**7**	Support for attending meetings and/or travel	☒ **None**	
**8**	Patents planned, issued or pending	☒ **None**	
**9**	Participation on a Data Safety Monitoring Board or Advisory Board	☒ **None**	
**10**	Leadership or fiduciary role in other board, society, committee or advocacy group, paid or unpaid	☒ **None**	

		Name all entities with whom you have this relationship or indicate none (add rows as needed)	Specifications/Comments (e.g., if payments were made to you or to your institution)
**11**	Stock or stock options	☒ **None**	
**12**	Receipt of equipment, materials, drugs, medical writing, gifts or other services	☒ **None**	
**13**	Other financial or non-financial interests	☒ **None**	

Please place an "X" next to the following statement to indicate your agreement:

☒ I certify that I have answered every question and have not altered the wording of any of the questions on this form.

ICMJE DISCLOSURE FORM

Date: 6/16/2024

Your Name: Gillian Daly

Manuscript Title: A common outcome set for trials in dementia with Lewy bodies (DLB COS)

Manuscript Number (if known): TRCI-D-25-00068R1

In the interest of transparency, we ask you to disclose all relationships/activities/interests listed below that are related to the content of your manuscript. "Related" means any relation with for-profit or not-for-profit third parties whose interests may be affected by the content of the manuscript. Disclosure represents a commitment to transparency and does not necessarily indicate a bias. If you are in doubt about whether to list a relationship/activity/interest, it is preferable that you do so.

The author's relationships/activities/interests should be defined broadly. For example, if your manuscript pertains to the epidemiology of hypertension, you should declare all relationships with manufacturers of antihypertensive medication, even if that medication is not mentioned in the manuscript.

In item #1 below, report all support for the work reported in this manuscript without time limit. For all other items, the time frame for disclosure is the past 36 months.

		Name all entities with whom you have this relationship or indicate none (add rows as needed)	Specifications/Comments (e.g., if payments were made to you or to your institution)
Time frame: Since the initial planning of the work			
1	All support for the present manuscript (e.g., funding, provision of study materials, medical writing, article processing charges, etc.) **No time limit for this item.**	☒ None	
			Click the tab key to add additional rows.
Time frame: past 36 months			
**2**	Grants or contracts from any entity (if not indicated in item #1 above).	☒ **None**	
**3**	Royalties or licenses	☒ **None**	

		Name all entities with whom you have this relationship or indicate none (add rows as needed)	Specifications/Comments (e.g., if payments were made to you or to your institution)
**4**	Consulting fees	☒ **None**	
**5**	Payment or honoraria for lectures, presentations, speakers bureaus, manuscript writing or educational events	☒ **None**	
**6**	Payment for expert testimony	☒ **None**	
**7**	Support for attending meetings and/or travel	☒ **None**	
**8**	Patents planned, issued or pending	☒ **None**	
**9**	Participation on a Data Safety Monitoring Board or Advisory Board	☒ **None**	
**10**	Leadership or fiduciary role in other board, society, committee or advocacy group, paid or unpaid	☒ **None**	

		Name all entities with whom you have this relationship or indicate none (add rows as needed)	Specifications/Comments (e.g., if payments were made to you or to your institution)
**11**	Stock or stock options	☒ **None**	
**12**	Receipt of equipment, materials, drugs, medical writing, gifts or other services	☒ **None**	
**13**	Other financial or non-financial interests	☒ **None**	

Please place an "X" next to the following statement to indicate your agreement:

☒ I certify that I have answered every question and have not altered the wording of any of the questions on this form.

ICMJE DISCLOSURE FORM

**Date:**	June 12th, 2025
**Your Name:**	Emily Kalfas
**Manuscript Title:**	A common outcome set for trials in dementia with Lewy bodies (DLB COS)
**Manuscript Number (if known):**	TRCI-D-25-00068R1

In the interest of transparency, we ask you to disclose all relationships/activities/interests listed below that are related to the content of your manuscript. "Related" means any relation with for-profit or not-for-profit third parties whose interests may be affected by the content of the manuscript. Disclosure represents a commitment to transparency and does not necessarily indicate a bias. If you are in doubt about whether to list a relationship/activity/interest, it is preferable that you do so.

The author's relationships/activities/interests should be defined broadly. For example, if your manuscript pertains to the epidemiology of hypertension, you should declare all relationships with manufacturers of antihypertensive medication, even if that medication is not mentioned in the manuscript.

In item #1 below, report all support for the work reported in this manuscript without time limit. For all other items, the time frame for disclosure is the past 36 months.

		**Name all entities with whom you have this relationship or indicate none (add rows as needed)**	**Specifications/Comments (e.g., if payments were made to you or to your institution)**
		**Time frame: Since the initial planning of the work**	
**1**	All support for the present manuscript (e.g., funding, provision of study materials, medical writing, article processing charges, etc.) **No time limit for this item.**	☑ **None**<br>Click the tab key to add additional rows.	
		**Time frame: past 36 months**	
**2**	Grants or contracts from any entity (if not indicated in item #1 above).	☑ **None**	
**3**	Royalties or licenses	☑ **None**	

		Name all entities with whom you have this relationship or indicate none (add rows as needed)	Specifications/Comments (e.g., if payments were made to you or to your institution)
**4**	Consulting fees	☑ **None**	
**5**	Payment or honoraria for lectures, presentations, speakers bureaus, manuscript writing or educational events	☑ **None**	
**6**	Payment for expert testimony	☑ **None**	
**7**	Support for attending meetings and/or travel	☑ **None**	
**8**	Patents planned, issued or pending	☑ **None**	
**9**	Participation on a Data Safety Monitoring Board or Advisory Board	☑ **None**	
**10**	Leadership or fiduciary role in other board, society, committee or advocacy group, paid or unpaid	☑ **None**	

		Name all entities with whom you have this relationship or indicate none (add rows as needed)	Specifications/Comments (e.g., if payments were made to you or to your institution)
**11**	Stock or stock options	☑ **None**	
**12**	Receipt of equipment, materials, drugs, medical writing, gifts or other services	☑ **None**	
**13**	Other financial or non-financial interests	☑ **None**	

Please place an "X" next to the following statement to indicate your agreement:

X I certify that I have answered every question and have not altered the wording of any of the questions on this form.

ICMJE DISCLOSURE FORM

**Date:**	5/30/2025
**Your Name:**	Irina Kinchin
**Manuscript Title:**	A common outcome set for trials in dementia with Lewy bodies (DLB COS)
**Manuscript Number (if known):**	TRCI-D-25-00068R1

In the interest of transparency, we ask you to disclose all relationships/activities/interests listed below that are related to the content of your manuscript. "Related" means any relation with for-profit or not-for-profit third parties whose interests may be affected by the content of the manuscript. Disclosure represents a commitment to transparency and does not necessarily indicate a bias. If you are in doubt about whether to list a relationship/activity/interest, it is preferable that you do so.

The author's relationships/activities/interests should be defined broadly. For example, if your manuscript pertains to the epidemiology of hypertension, you should declare all relationships with manufacturers of antihypertensive medication, even if that medication is not mentioned in the manuscript.

In item #1 below, report all support for the work reported in this manuscript without time limit. For all other items, the time frame for disclosure is the past 36 months.

		**Name all entities with whom you have this relationship or indicate none (add rows as needed)**	**Specifications/Comments (e.g., if payments were made to you or to your institution)**
**Time frame: Since the initial planning of the work**			
1	All support for the present manuscript (e.g., funding, provision of study materials, medical writing, article processing charges, etc.) **No time limit for this item.**	☒ None	
			Click the tab key to add additional rows.
**Time frame: past 36 months**			
**2**	Grants or contracts from any entity (if not indicated in item #1 above).	☒ **None**	
**3**	Royalties or licenses	☒ **None**	

		Name all entities with whom you have this relationship or indicate none (add rows as needed)	Specifications/Comments (e.g., if payments were made to you or to your institution)
4	Consulting fees	☒ None	
5	Payment or honoraria for lectures, presentations, speakers bureaus, manuscript writing or educational events	☒ None	
6	Payment for expert testimony	☒ None	
7	Support for attending meetings and/or travel	☒ None	
8	Patents planned, issued or pending	☒ None	
9	Participation on a Data Safety Monitoring Board or Advisory Board	☒ None	
10	Leadership or fiduciary role in other board, society, committee or advocacy group, paid or unpaid	☒ None	

		Name all entities with whom you have this relationship or indicate none (add rows as needed)	Specifications/Comments (e.g., if payments were made to you or to your institution)
**11**	Stock or stock options	☒ **None**	
**12**	Receipt of equipment, materials, drugs, medical writing, gifts or other services	☒ **None**	
**13**	Other financial or non-financial interests	☒ **None**	

Please place an "X" next to the following statement to indicate your agreement:

☒ I certify that I have answered every question and have not altered the wording of any of the questions on this form.

ICMJE DISCLOSURE FORM

**Date:**	23/06/2025
**Your Name:**	Professor Dag Aarsland
**Manuscript Title:**	A common outcome set for trials in dementia with Lewy bodies (DLB COS)
**Manuscript Number (if known):**	TRCI-D-25-00068R1

In the interest of transparency, we ask you to disclose all relationships/activities/interests listed below that are related to the content of your manuscript. "Related" means any relation with for-profit or not-for-profit third parties whose interests may be affected by the content of the manuscript. Disclosure represents a commitment to transparency and does not necessarily indicate a bias. If you are in doubt about whether to list a relationship/activity/interest, it is preferable that you do so.

The author's relationships/activities/interests should be defined broadly. For example, if your manuscript pertains to the epidemiology of hypertension, you should declare all relationships with manufacturers of antihypertensive medication, even if that medication is not mentioned in the manuscript.

In item #1 below, report all support for the work reported in this manuscript without time limit. For all other items, the time frame for disclosure is the past 36 months.

		**Name all entities with whom you have this relationship or indicate none (add rows as needed)**	**Specifications/Comments (e.g., if payments were made to you or to your institution)**
		**Time frame: Since the initial planning of the work**	
1	All support for the present manuscript (e.g., funding, provision of study materials, medical writing, article processing charges, etc.) **No time limit for this item.**	☒ None	
			Click the tab key to add additional rows.
		**Time frame: past 36 months**	
**2**	Grants or contracts from any entity (if not indicated in item #1 above).	☒ **None**	
**3**	Royalties or licenses	☒ **None**	

		Name all entities with whom you have this relationship or indicate none (add rows as needed)	Specifications/Comments (e.g., if payments were made to you or to your institution)
4	Consulting fees	☒ None	
		Acadia	
GSK	Payment or honoraria for lectures, presentations, speakers bureaus, manuscript writing or educational events	☒ None	
		GSK	
		Eisai	
6	Payment for expert testimony	☒ None	
7	Support for attending meetings and/or travel	☒ None	
8	Patents planned, issued or pending	☒ None	
9	Participation on a Data Safety Monitoring Board or Advisory Board	☐ None	
		BioArctic	
		Roche Diagnostics	
		Eli Lilly UK	
10	Leadership or fiduciary role in other board, society, committee or advocacy group, paid or unpaid	☒ None	

		Name all entities with whom you have this relationship or indicate none (add rows as needed)	Specifications/Comments (e.g., if payments were made to you or to your institution)
**11**	Stock or stock options	☒ **None**	
**12**	Receipt of equipment, materials, drugs, medical writing, gifts or other services	☐ **None**	
		Roche Diagnostics	
**13**	Other financial or non-financial interests	☒ **None**	

Please place an "X" next to the following statement to indicate your agreement:

☐ I certify that I have answered every question and have not altered the wording of any of the questions on this form.

ICMJE DISCLOSURE FORM

**Date:**	5/30/2025
**Your Name:**	Kenneth Greaney
**Manuscript Title:**	A common outcome set for trials in dementia with Lewy bodies (DLB COS)
**Manuscript Number (if known):**	TRCI-D-25-00068R1

In the interest of transparency, we ask you to disclose all relationships/activities/interests listed below that are related to the content of your manuscript. "Related" means any relation with for-profit or not-for-profit third parties whose interests may be affected by the content of the manuscript. Disclosure represents a commitment to transparency and does not necessarily indicate a bias. If you are in doubt about whether to list a relationship/activity/interest, it is preferable that you do so.

The author's relationships/activities/interests should be defined broadly. For example, if your manuscript pertains to the epidemiology of hypertension, you should declare all relationships with manufacturers of antihypertensive medication, even if that medication is not mentioned in the manuscript.

In item #1 below, report all support for the work reported in this manuscript without time limit. For all other items, the time frame for disclosure is the past 36 months.

		**Name all entities with whom you have this relationship or indicate none (add rows as needed)**	**Specifications/Comments (e.g., if payments were made to you or to your institution)**
**Time frame: Since the initial planning of the work**			
1	All support for the present manuscript (e.g., funding, provision of study materials, medical writing, article processing charges, etc.) **No time limit for this item.**	☒ None	
			Click the tab key to add additional rows.
**Time frame: past 36 months**			
**2**	Grants or contracts from any entity (if not indicated in item #1 above).	☒ **None**	
**3**	Royalties or licenses	☒ **None**	

		Name all entities with whom you have this relationship or indicate none (add rows as needed)	Specifications/Comments (e.g., if payments were made to you or to your institution)
**4**	Consulting fees	☒ **None**	
**5**	Payment or honoraria for lectures, presentations, speakers bureaus, manuscript writing or educational events	☒ **None**	
**6**	Payment for expert testimony	☒ **None**	
**7**	Support for attending meetings and/or travel	☒ **None**	
**8**	Patents planned, issued or pending	☒ **None**	
**9**	Participation on a Data Safety Monitoring Board or Advisory Board	☒ **None**	
**10**	Leadership or fiduciary role in other board, society, committee or advocacy group, paid or unpaid	☒ **None**	

		Name all entities with whom you have this relationship or indicate none (add rows as needed)	Specifications/Comments (e.g., if payments were made to you or to your institution)
**11**	Stock or stock options	☒ **None**	
**12**	Receipt of equipment, materials, drugs, medical writing, gifts or other services	☒ **None**	
**13**	Other financial or non-financial interests	☒ **None**	

Please place an "X" next to the following statement to indicate your agreement:

☒ I certify that I have answered every question and have not altered the wording of any of the questions on this form.

ICMJE DISCLOSURE FORM

**Date:**	18/06/2025
**Your Name:**	Emilia Grycuk
**Manuscript Title:**	A common outcome set for trials in dementia with Lewy bodies (DLB COS)
**Manuscript Number (if known):**	TRCI-D-25-00068R1

In the interest of transparency, we ask you to disclose all relationships/activities/interests listed below that are related to the content of your manuscript. "Related" means any relation with for-profit or not-for-profit third parties whose interests may be affected by the content of the manuscript. Disclosure represents a commitment to transparency and does not necessarily indicate a bias. If you are in doubt about whether to list a relationship/activity/interest, it is preferable that you do so.

The author's relationships/activities/interests should be defined broadly. For example, if your manuscript pertains to the epidemiology of hypertension, you should declare all relationships with manufacturers of antihypertensive medication, even if that medication is not mentioned in the manuscript.

In item #1 below, report all support for the work reported in this manuscript without time limit. For all other items, the time frame for disclosure is the past 36 months.

		**Name all entities with whom you have this relationship or indicate none (add rows as needed)**	**Specifications/Comments (e.g., if payments were made to you or to your institution)**
**Time frame: Since the initial planning of the work**			
1	All support for the present manuscript (e.g., funding, provision of study materials, medical writing, article processing charges, etc.) No time limit for this item.	☒ None	
			Click the tab key to add additional rows.
**Time frame: past 36 months**			
**2**	Grants or contracts from any entity (if not indicated in item #1 above).	☒ **None**	
**3**	Royalties or licenses	☒ **None**	

		Name all entities with whom you have this relationship or indicate none (add rows as needed)	Specifications/Comments (e.g., if payments were made to you or to your institution)
**4**	Consulting fees	☒ **None**	
**5**	Payment or honoraria for lectures, presentations, speakers bureaus, manuscript writing or educational events	☒ **None**	
**6**	Payment for expert testimony	☒ **None**	
**7**	Support for attending meetings and/or travel	☒ **None**	
**8**	Patents planned, issued or pending	☒ **None**	
**9**	Participation on a Data Safety Monitoring Board or Advisory Board	☒ **None**	
**10**	Leadership or fiduciary role in other board, society, committee or advocacy group, paid or unpaid	☒ **None**	

		Name all entities with whom you have this relationship or indicate none (add rows as needed)	Specifications/Comments (e.g., if payments were made to you or to your institution)
**11**	Stock or stock options	☒ **None**	
**12**	Receipt of equipment, materials, drugs, medical writing, gifts or other services	☒ **None**	
**13**	Other financial or non-financial interests	☒ **None**	

Please place an "X" next to the following statement to indicate your agreement:

☒ I certify that I have answered every question and have not altered the wording of any of the questions on this form.

ICMJE DISCLOSURE FORM

Date: 6/11/2025

Your Name: Dr. Ann-Kristin Folkerts

Manuscript Title: A common outcome set for trials in dementia with Lewy bodies (DLB COS)

Manuscript Number (if known): TRCI-D-25-00068R1

In the interest of transparency, we ask you to disclose all relationships/activities/interests listed below that are related to the content of your manuscript. "Related" means any relation with for-profit or not-for-profit third parties whose interests may be affected by the content of the manuscript. Disclosure represents a commitment to transparency and does not necessarily indicate a bias. If you are in doubt about whether to list a relationship/activity/interest, it is preferable that you do so.

The author's relationships/activities/interests should be defined broadly. For example, if your manuscript pertains to the epidemiology of hypertension, you should declare all relationships with manufacturers of antihypertensive medication, even if that medication is not mentioned in the manuscript.

In item #1 below, report all support for the work reported in this manuscript without time limit. For all other items, the time frame for disclosure is the past 36 months.

		**Name all entities with whom you have this relationship or indicate none (add rows as needed)**	**Specifications/Comments (e.g., if payments were made to you or to your institution)**
**Time frame: Since the initial planning of the work**			
1	All support for the present manuscript (e.g., funding, provision of study materials, medical writing, article processing charges, etc.) **No time limit for this item.**	☒ None	
			Click the tab key to add additional rows.
**Time frame: past 36 months**			
**2**	Grants or contracts from any entity (if not indicated in item #1 above).	☒ **None**	
**3**	Royalties or licenses	☒ **None**	

		Name all entities with whom you have this relationship or indicate none (add rows as needed)	Specifications/Comments (e.g., if payments were made to you or to your institution)
**4**	Consulting fees	☒ **None**	
**5**	Payment or honoraria for lectures, presentations, speakers bureaus, manuscript writing or educational events	☒ **None**	
**6**	Payment for expert testimony	☒ **None**	
**7**	Support for attending meetings and/or travel	☒ **None**	
**8**	Patents planned, issued or pending	☒ **None**	
**9**	Participation on a Data Safety Monitoring Board or Advisory Board	☒ **None**	
**10**	Leadership or fiduciary role in other board, society, committee or advocacy group, paid or unpaid	☒ **None**	

		Name all entities with whom you have this relationship or indicate none (add rows as needed)	Specifications/Comments (e.g., if payments were made to you or to your institution)
**11**	Stock or stock options	☒ **None**	
**12**	Receipt of equipment, materials, drugs, medical writing, gifts or other services	☒ **None**	
**13**	Other financial or non-financial interests	☒ **None**	

Please place an "X" next to the following statement to indicate your agreement:

☒ I certify that I have answered every question and have not altered the wording of any of the questions on this form.

ICMJE DISCLOSURE FORM

**Date:**	6/11/2025
**Your Name:**	Elke Kalbe
**Manuscript Title:**	A common outcome set for trials in dementia with Lewy bodies (DLB COS)
**Manuscript Number (if known):**	TRCI-D-25-00068R1

In the interest of transparency, we ask you to disclose all relationships/activities/interests listed below that are related to the content of your manuscript. "Related" means any relation with for-profit or not-for-profit third parties whose interests may be affected by the content of the manuscript. Disclosure represents a commitment to transparency and does not necessarily indicate a bias. If you are in doubt about whether to list a relationship/activity/interest, it is preferable that you do so.

The author's relationships/activities/interests should be defined broadly. For example, if your manuscript pertains to the epidemiology of hypertension, you should declare all relationships with manufacturers of antihypertensive medication, even if that medication is not mentioned in the manuscript.

In item #1 below, report all support for the work reported in this manuscript without time limit. For all other items, the time frame for disclosure is the past 36 months.

		**Name all entities with whom you have this relationship or indicate none (add rows as needed)**	**Specifications/Comments (e.g., if payments were made to you or to your institution)**
**Time frame: Since the initial planning of the work**			
1	All support for the present manuscript (e.g., funding, provision of study materials, medical writing, article processing charges, etc.) **No time limit for this item.**	☒ None	
			Click the tab key to add additional rows.
**Time frame: past 36 months**			
**2**	Grants or contracts from any entity (if not indicated in item #1 above).	☒ **None**	
**3**	Royalties or licenses	☒ **None**	

		Name all entities with whom you have this relationship or indicate none (add rows as needed)	Specifications/Comments (e.g., if payments were made to you or to your institution)
**4**	Consulting fees	☒ **None**	
**5**	Payment or honoraria for lectures, presentations, speakers bureaus, manuscript writing or educational events	☒ **None**	
**6**	Payment for expert testimony	☒ **None**	
**7**	Support for attending meetings and/or travel	☒ **None**	
**8**	Patents planned, issued or pending	☒ **None**	
**9**	Participation on a Data Safety Monitoring Board or Advisory Board	☒ **None**	
**10**	Leadership or fiduciary role in other board, society, committee or advocacy group, paid or unpaid	☒ **None**	

		Name all entities with whom you have this relationship or indicate none (add rows as needed)	Specifications/Comments (e.g., if payments were made to you or to your institution)
**11**	Stock or stock options	☒ **None**	
**12**	Receipt of equipment, materials, drugs, medical writing, gifts or other services	☒ **None**	
**13**	Other financial or non-financial interests	☒ **None**	

Please place an "X" next to the following statement to indicate your agreement:

☒ I certify that I have answered every question and have not altered the wording of any of the questions on this form.

ICMJE DISCLOSURE FORM

**Date:**	5/29/2025
**Your Name:**	Federico Rodriguez-Porcel
**Manuscript Title:**	A common outcome set for trials in dementia with Lewy bodies (DLB COS)
**Manuscript Number (if known):**	TRCI-D-25-00068R1

In the interest of transparency, we ask you to disclose all relationships/activities/interests listed below that are related to the content of your manuscript. “Related” means any relation with for-profit or not-for-profit third parties whose interests may be affected by the content of the manuscript. Disclosure represents a commitment to transparency and does not necessarily indicate a bias. If you are in doubt about whether to list a relationship/activity/interest, it is preferable that you do so.

The author’s relationships/activities/interests should be defined broadly. For example, if your manuscript pertains to the epidemiology of hypertension, you should declare all relationships with manufacturers of antihypertensive medication, even if that medication is not mentioned in the manuscript.

In item #1 below, report all support for the work reported in this manuscript without time limit. For all other items, the time frame for disclosure is the past 36 months.

		**Name all entities with whom you have this relationship or indicate none (add rows as needed)**	**Specifications/Comments (e.g., if payments were made to you or to your institution)**
		Time frame: Since the initial planning of the work	
1	All support for the present manuscript (e.g., funding, provision of study materials, medical writing, article processing charges, etc.) **No time limit for this item.**	☒ None	
			Click the tab key to add additional rows.
		Time frame: past 36 months	
**2**	Grants or contracts from any entity (if not indicated in item #1 above).	☐ **None**	
		NIH-R21DC019749	PI
**3**	Royalties or licenses	☐ **None**	
		Cambridge University Press	Royalties from book

		Name all entities with whom you have this relationship or indicate none (add rows as needed)	Specifications/Comments (e.g., if payments were made to you or to your institution)
**4**	Consulting fees	☒ **None**	
**5**	Payment or honoraria for lectures, presentations, speakers bureaus, manuscript writing or educational events	☐ **None**	
		Movement Disorders Society	Course director
**6**	Payment for expert testimony	☒ **None**	
**7**	Support for attending meetings and/or travel	☒ **None**	
**8**	Patents planned, issued or pending	☒ **None**	
**9**	Participation on a Data Safety Monitoring Board or Advisory Board	☒ **None**	
**10**	Leadership or fiduciary role in other board, society, committee or advocacy group, paid or unpaid	☒ **None**	

		Name all entities with whom you have this relationship or indicate none (add rows as needed)	Specifications/Comments (e.g., if payments were made to you or to your institution)
**11**	Stock or stock options	☒ **None**	
**12**	Receipt of equipment, materials, drugs, medical writing, gifts or other services	☒ **None**	
**13**	Other financial or non-financial interests	☒ **None**	

Please place an "X" next to the following statement to indicate your agreement:

☒ I certify that I have answered every question and have not altered the wording of any of the questions on this form.

ICMJE DISCLOSURE FORM

Date: 5/30/2025

Your Name: Ian Saldanha

Manuscript Title: A common outcome set for trials in dementia with Lewy bodies (DLB COS)

Manuscript Number (if known): TRCI-D-25-00068R1

In the interest of transparency, we ask you to disclose all relationships/activities/interests listed below that are related to the content of your manuscript. "Related" means any relation with for-profit or not-for-profit third parties whose interests may be affected by the content of the manuscript. Disclosure represents a commitment to transparency and does not necessarily indicate a bias. If you are in doubt about whether to list a relationship/activity/interest, it is preferable that you do so.

The author's relationships/activities/interests should be defined broadly. For example, if your manuscript pertains to the epidemiology of hypertension, you should declare all relationships with manufacturers of antihypertensive medication, even if that medication is not mentioned in the manuscript.

In item #1 below, report all support for the work reported in this manuscript without time limit. For all other items, the time frame for disclosure is the past 36 months.

		Name all entities with whom you have this relationship or indicate none (add rows as needed)	Specifications/Comments (e.g., if payments were made to you or to your institution)
Time frame: Since the initial planning of the work			
1	All support for the present manuscript (e.g., funding, provision of study materials, medical writing, article processing charges, etc.) **No time limit for this item.**	☒ None	
			Click the tab key to add additional rows.
Time frame: past 36 months			
**2**	Grants or contracts from any entity (if not indicated in item #1 above).	☒ **None**	
**3**	Royalties or licenses	☒ **None**	

		Name all entities with whom you have this relationship or indicate none (add rows as needed)	Specifications/Comments (e.g., if payments were made to you or to your institution)
**4**	Consulting fees	☒ **None**	
**5**	Payment or honoraria for lectures, presentations, speakers bureaus, manuscript writing or educational events	☒ **None**	
**6**	Payment for expert testimony	☒ **None**	
**7**	Support for attending meetings and/or travel	☒ **None**	
**8**	Patents planned, issued or pending	☒ **None**	
**9**	Participation on a Data Safety Monitoring Board or Advisory Board	☒ **None**	
**10**	Leadership or fiduciary role in other board, society, committee or advocacy group, paid or unpaid	☒ **None**	

		Name all entities with whom you have this relationship or indicate none (add rows as needed)	Specifications/Comments (e.g., if payments were made to you or to your institution)
**11**	Stock or stock options	☒ **None**	
**12**	Receipt of equipment, materials, drugs, medical writing, gifts or other services	☒ **None**	
**13**	Other financial or non-financial interests	☒ **None**	

Please place an "X" next to the following statement to indicate your agreement:

☒ I certify that I have answered every question and have not altered the wording of any of the questions on this form.

ICMJE DISCLOSURE FORM

**Date:**	5/30/2025
**Your Name:**	Valerie Smith
**Manuscript Title:**	A common outcome set for trials in dementia with Lewy bodies (DLB COS)
**Manuscript Number (if known):**	TRCI-D-25-00068R1

In the interest of transparency, we ask you to disclose all relationships/activities/interests listed below that are related to the content of your manuscript. "Related" means any relation with for-profit or not-for-profit third parties whose interests may be affected by the content of the manuscript. Disclosure represents a commitment to transparency and does not necessarily indicate a bias. If you are in doubt about whether to list a relationship/activity/interest, it is preferable that you do so.

The author's relationships/activities/interests should be defined broadly. For example, if your manuscript pertains to the epidemiology of hypertension, you should declare all relationships with manufacturers of antihypertensive medication, even if that medication is not mentioned in the manuscript.

In item #1 below, report all support for the work reported in this manuscript without time limit. For all other items, the time frame for disclosure is the past 36 months.

		**Name all entities with whom you have this relationship or indicate none (add rows as needed)**	**Specifications/Comments (e.g., if payments were made to you or to your institution)**
**Time frame: Since the initial planning of the work**			
**1**	All support for the present manuscript (e.g., funding, provision of study materials, medical writing, article processing charges, etc.) **No time limit for this item.**	☒ None	
			Click the tab key to add additional rows.
**Time frame: past 36 months**			
**2**	Grants or contracts from any entity (if not indicated in item #1 above).	☒ **None**	
**3**	Royalties or licenses	☒ **None**	

		Name all entities with whom you have this relationship or indicate none (add rows as needed)	Specifications/Comments (e.g., if payments were made to you or to your institution)
**4**	Consulting fees	☒ **None**	
**5**	Payment or honoraria for lectures, presentations, speakers bureaus, manuscript writing or educational events	☒ **None**	
**6**	Payment for expert testimony	☒ **None**	
**7**	Support for attending meetings and/or travel	☒ **None**	
**8**	Patents planned, issued or pending	☒ **None**	
**9**	Participation on a Data Safety Monitoring Board or Advisory Board	☒ **None**	
**10**	Leadership or fiduciary role in other board, society, committee or advocacy group, paid or unpaid	☒ **None**	

		Name all entities with whom you have this relationship or indicate none (add rows as needed)	Specifications/Comments (e.g., if payments were made to you or to your institution)
**11**	Stock or stock options	☒ **None**	
**12**	Receipt of equipment, materials, drugs, medical writing, gifts or other services	☒ **None**	
**13**	Other financial or non-financial interests	☒ **None**	

Please place an "X" next to the following statement to indicate your agreement:

☒ I certify that I have answered every question and have not altered the wording of any of the questions on this form.

ICMJE DISCLOSURE FORM

Date: 5/31/2025

Your Name: John-Paul Taylor

Manuscript Title: A common outcome set for trials in dementia with Lewy bodies (DLB COS)

Manuscript Number (if known): TRCI-D-25-00068R1

In the interest of transparency, we ask you to disclose all relationships/activities/interests listed below that are related to the content of your manuscript. "Related" means any relation with for-profit or not-for-profit third parties whose interests may be affected by the content of the manuscript. Disclosure represents a commitment to transparency and does not necessarily indicate a bias. If you are in doubt about whether to list a relationship/activity/interest, it is preferable that you do so.

The author's relationships/activities/interests should be defined broadly. For example, if your manuscript pertains to the epidemiology of hypertension, you should declare all relationships with manufacturers of antihypertensive medication, even if that medication is not mentioned in the manuscript.

In item #1 below, report all support for the work reported in this manuscript without time limit. For all other items, the time frame for disclosure is the past 36 months.

		**Name all entities with whom you have this relationship or indicate none (add rows as needed)**	**Specifications/Comments (e.g., if payments were made to you or to your institution)**
		Time frame: Since the initial planning of the work	
1	All support for the present manuscript (e.g., funding, provision of study materials, medical writing, article processing charges, etc.) **No time limit for this item.**	☒ None	
			Click the tab key to add additional rows.
		Time frame: past 36 months	
2	Grants or contracts from any entity (if not indicated in item #1 above).	☐ **None**	
		NIHR Newcastle Biomedical Research Centre	J-P Taylor is supported by Newcastle BRC
3	Royalties or licenses	☒ **None**	

		Name all entities with whom you have this relationship or indicate none (add rows as needed)	Specifications/Comments (e.g., if payments were made to you or to your institution)
**4**	Consulting fees	☒ **None**	
**5**	Payment or honoraria for lectures, presentations, speakers bureaus, manuscript writing or educational events	☒ **None**	
**6**	Payment for expert testimony	☒ **None**	
**7**	Support for attending meetings and/or travel	☒ **None**	
**8**	Patents planned, issued or pending	☒ **None**	
**9**	Participation on a Data Safety Monitoring Board or Advisory Board	☒ **None**	
**10**	Leadership or fiduciary role in other board, society, committee or advocacy group, paid or unpaid	☒ **None**	

		Name all entities with whom you have this relationship or indicate none (add rows as needed)	Specifications/Comments (e.g., if payments were made to you or to your institution)
**11**	Stock or stock options	☒ **None**	
**12**	Receipt of equipment, materials, drugs, medical writing, gifts or other services	☒ **None**	
**13**	Other financial or non-financial interests	☒ **None**	

Please place an "X" next to the following statement to indicate your agreement:

☒ I certify that I have answered every question and have not altered the wording of any of the questions on this form.

ICMJE DISCLOSURE FORM

Date: 6/10/2025

Your Name: Rachel Thompson

Manuscript Title: A common outcome set for trials in dementia with Lewy bodies (DLB COS)

Manuscript Number (if known): TRCI-D-25-00068R1

In the interest of transparency, we ask you to disclose all relationships/activities/interests listed below that are related to the content of your manuscript. "Related" means any relation with for-profit or not-for-profit third parties whose interests may be affected by the content of the manuscript. Disclosure represents a commitment to transparency and does not necessarily indicate a bias. If you are in doubt about whether to list a relationship/activity/interest, it is preferable that you do so.

The author's relationships/activities/interests should be defined broadly. For example, if your manuscript pertains to the epidemiology of hypertension, you should declare all relationships with manufacturers of antihypertensive medication, even if that medication is not mentioned in the manuscript.

In item #1 below, report all support for the work reported in this manuscript without time limit. For all other items, the time frame for disclosure is the past 36 months.

		Name all entities with whom you have this relationship or indicate none (add rows as needed)	Specifications/Comments (e.g., if payments were made to you or to your institution)
Time frame: Since the initial planning of the work			
1	All support for the present manuscript (e.g., funding, provision of study materials, medical writing, article processing charges, etc.) **No time limit for this item.**	☒ None	
			Click the tab key to add additional rows.
Time frame: past 36 months			
**2**	Grants or contracts from any entity (if not indicated in item #1 above).	☒ **None**	
**3**	Royalties or licenses	☒ **None**	

		Name all entities with whom you have this relationship or indicate none (add rows as needed)	Specifications/Comments (e.g., if payments were made to you or to your institution)
**4**	Consulting fees	☒ **None**	
**5**	Payment or honoraria for lectures, presentations, speakers bureaus, manuscript writing or educational events	☒ **None**	
		Neurology Academy	Payment for presentations
**6**	Payment for expert testimony	☒ **None**	
**7**	Support for attending meetings and/or travel	☒ **None**	
**8**	Patents planned, issued or pending	☒ **None**	
**9**	Participation on a Data Safety Monitoring Board or Advisory Board	☒ **None**	
**10**	Leadership or fiduciary role in other board, society, committee or advocacy group, paid or unpaid	☐ **None**	
		The Lewy Body Society (member of advisory committee)	Remuneration as part of paid role

		Name all entities with whom you have this relationship or indicate none (add rows as needed)	Specifications/Comments (e.g., if payments were made to you or to your institution)
**11**	Stock or stock options	☒ **None**	
**12**	Receipt of equipment, materials, drugs, medical writing, gifts or other services	☒ **None**	
**13**	Other financial or non-financial interests	☒ **None**	

Please place an "X" next to the following statement to indicate your agreement:

☒ I certify that I have answered every question and have not altered the wording of any of the questions on this form.

ICMJE DISCLOSURE FORM

**Date:**	06/02/2025
**Your Name:**	Kathryn Wyman-Chick
**Manuscript Title:**	A common outcome set for trials in dementia with Lewy bodies (DLB COS)
**Manuscript Number (if known):**	TRCI-D-25-00068R1

In the interest of transparency, we ask you to disclose all relationships/activities/interests listed below that are related to the content of your manuscript. "Related" means any relation with for-profit or not-for-profit third parties whose interests may be affected by the content of the manuscript. Disclosure represents a commitment to transparency and does not necessarily indicate a bias. If you are in doubt about whether to list a relationship/activity/interest, it is preferable that you do so.

The author's relationships/activities/interests should be defined broadly. For example, if your manuscript pertains to the epidemiology of hypertension, you should declare all relationships with manufacturers of antihypertensive medication, even if that medication is not mentioned in the manuscript.

In item #1 below, report all support for the work reported in this manuscript without time limit. For all other items, the time frame for disclosure is the past 36 months.

		**Name all entities with whom you have this relationship or indicate none (add rows as needed)**	**Specifications/Comments (e.g., if payments were made to you or to your institution)**
**Time frame: Since the initial planning of the work**			
**1**	All support for the present manuscript (e.g., funding, provision of study materials, medical writing, article processing charges, etc.) **No time limit for this item.**	☒ **None**	
			Click the tab key to add additional rows.
**Time frame: past 36 months**			
**2**	Grants or contracts from any entity (if not indicated in item #1 above).	☐ **None**	
		NIH/NIA R21AG074368 – Principal Investigator	Paid to institution
		Lewy Body Dementia Association Research Center of Excellence – Principal Investigator	Paid to institution

		Name all entities with whom you have this relationship or indicate none (add rows as needed)	Specifications/Comments (e.g., if payments were made to you or to your institution)
3	Royalties or licenses	☐ None	
		Oxford University Press – Textbook	Paid to me
4	Consulting fees	☒ None	
5	Payment or honoraria for lectures, presentations, speakers bureaus, manuscript writing or educational events	☐ None	
		American Academy of Clinical Neuropsychology Conference Honorarium	Paid to me
		University of Tennesee Annual Symposium on Alzheimers and Dementia Honorarium	Paid to me
		Henry Stewart Talks LTD Lecture Honorarium	Paid to me
6	Payment for expert testimony	☒ None	
7	Support for attending meetings and/or travel	☒ None	
8	Patents planned, issued or pending	☒ None	
9	Participation on a Data Safety	☒ None	

		Name all entities with whom you have this relationship or indicate none (add rows as needed)	Specifications/Comments (e.g., if payments were made to you or to your institution)
	Monitoring Board or Advisory Board		
10	Leadership or fiduciary role in other board, society, committee or advocacy group, paid or unpaid	☒ None	
11	Stock or stock options	☒ None	
12	Receipt of equipment, materials, drugs, medical writing, gifts or other services	☒ None	
13	Other financial or non-financial interests	☒ None	

Please place an "X" next to the following statement to indicate your agreement:

☒ I certify that I have answered every question and have not altered the wording of any of the questions on this form.

ICMJE DISCLOSURE FORM

**Date:**	5/29/2025
**Your Name:**	Iracema Leroi
**Manuscript Title:**	A common outcome set for trials in dementia with Lewy bodies (DLB COS)TRCI-D-
**Manuscript Number (if known):**	25-00068R1

In the interest of transparency, we ask you to disclose all relationships/activities/interests listed below that are related to the content of your manuscript. "Related" means any relation with for-profit or not-for-profit third parties whose interests may be affected by the content of the manuscript. Disclosure represents a commitment to transparency and does not necessarily indicate a bias. If you are in doubt about whether to list a relationship/activity/interest, it is preferable that you do so.

The author's relationships/activities/interests should be defined broadly. For example, if your manuscript pertains to the epidemiology of hypertension, you should declare all relationships with manufacturers of antihypertensive medication, even if that medication is not mentioned in the manuscript.

In item #1 below, report all support for the work reported in this manuscript without time limit. For all other items, the time frame for disclosure is the past 36 months.

		Name all entities with whom you have this relationship or indicate none (add rows as needed)	Specifications/Comments (e.g., if payments were made to you or to your institution)
**Time frame: Since the initial planning of the work**			
1	All support for the present manuscript (e.g., funding, provision of study materials, medical writing, article processing charges, etc.) **No time limit for this item.**	☒ None	
			Click the tab key to add additional rows.
**Time frame: past 36 months**			
**2**	Grants or contracts from any entity (if not indicated in item #1 above).	☐ **None**	
		HRB APRO 48 month research grant for EMERALD LEwy	HRB-CTN Dementia Trials Ireland
		HRB KTA support	HRB Disssemination grant
		ESTHER Ireland, International Global Network fund	
**3**	Royalties or licenses	☒ **None**	

		Name all entities with whom you have this relationship or indicate none (add rows as needed)	Specifications/Comments (e.g., if payments were made to you or to your institution)
**4**	Consulting fees	☐ **None**	
		Eisai advisory board (unrelated to manuscript)	
		NovoNordiks consulting fee (unrelated)	
**5**	Payment or honoraria for lectures, presentations, speakers bureaus, manuscript writing or educational events	☐ **None**	
		Bial speaker fee for single lecture	
**6**	Payment for expert testimony	☒ **None**	
**7**	Support for attending meetings and/or travel	☒ **None**	
**8**	Patents planned, issued or pending	☒ **None**	
**9**	Participation on a Data Safety Monitoring Board or Advisory Board	☐ **None**	
		TSC for investigator led CLEVER study (Wellcome Trust)	
		TSC for investigator led ACT RCT study (UK NIHR funded)	
**10**	Leadership or fiduciary role in other board, society, committee or advocacy group, paid or unpaid	☐ **None**	
		Governing board, charity, Lewy body IReland	
		Governing board, charity Walk the Talk for Dementia	

		Name all entities with whom you have this relationship or indicate none (add rows as needed)	Specifications/Comments (e.g., if payments were made to you or to your institution)
**11**	Stock or stock options	☒ **None**	
**12**	Receipt of equipment, materials, drugs, medical writing, gifts or other services	☒ **None**	
**13**	Other financial or non-financial interests	☒ **None**	

Please place an "X" next to the following statement to indicate your agreement:

☒ I certify that I have answered every question and have not altered the wording of any of the questions on this form.

ICMJE DISCLOSURE FORM

**Date:**	6/24/2025
**Your Name:**	DLB COS Delphi Group
**Manuscript Title:**	A common outcome set for trials in dementia with Lewy bodies (DLB COS)
**Manuscript Number (if known):**	TRCI-D-25-00068R1

In the interest of transparency, we ask you to disclose all relationships/activities/interests listed below that are related to the content of your manuscript. “Related” means any relation with for-profit or not-for-profit third parties whose interests may be affected by the content of the manuscript. Disclosure represents a commitment to transparency and does not necessarily indicate a bias. If you are in doubt about whether to list a relationship/activity/interest, it is preferable that you do so.

The author’s relationships/activities/interests should be defined broadly. For example, if your manuscript pertains to the epidemiology of hypertension, you should declare all relationships with manufacturers of antihypertensive medication, even if that medication is not mentioned in the manuscript.

In item #1 below, report all support for the work reported in this manuscript without time limit. For all other items, the time frame for disclosure is the past 36 months.

		**Name all entities with whom you have this relationship or indicate none (add rows as needed)**	**Specifications/Comments (e.g., if payments were made to you or to your institution)**
		Time frame: Since the initial planning of the work	
1	All support for the present manuscript (e.g., funding, provision of study materials, medical writing, article processing charges, etc.) **No time limit for this item.**	☐ None	
		Paul C Donaghy - Medical Research Council	Grant number MR/W000229/1
		Paul C Donaghy - NIHR Newcastle Biomedical Research Centre	
		David Irwin - NIH	Research funding to institution
		David Irwin - Penn IOA	Research funding to institution
		Simon Lewis - NHMRC Investigator Grant (#1195830)	
		Sonja W Scholz - NIH	Intramural Research Program
		Time frame: past 36 months	
2	Grants or contracts from any entity (if not indicated in item #1 above).	☐ **None**	
		Louise Allan - National Institute of Health and Care Research, UK	
		Louise Allan - Alzheimer’s Society, UK	
		Louise Allan - Medical Research Council, UK	
		Laura Bonanni - Italian Ministry of Health (PNRR fund 2022 and 2023)	
		Laura Bonanni - Italian Ministry of University (PRIN 2022)	
		Andrea Bozoki - EIP Pharma (now Cervomed)	
		Andrea Bozoki - Cognition Therapeutics	
		Andrea Bozoki - Alector Pharma	
		Andrea Bozoki - Transposon Therapeutics	
		Andrea Bozoki - North Carolina Dept of Health & Human Services	

		Name all entities with whom you have this relationship or indicate none (add rows as needed)	Specifications/Comments (e.g., if payments were made to you or to your institution)

Andrea Bozoki - Kinetix (now PetauriKinect)	
David Bradley - Health Research Board Ireland grant APRO-2023-013	
David Bradley - Health Research Board Ireland grant SDAP-2023-041	
Paul C Donaghy - GE Healthcare	
Paul C Donaghy - Michael J Fox Foundation	
Paul C Donaghy - Alzheimer's Society	
Paul C Donaghy - Alzheimer's Research UK	
Daniel Ferreira - Swedish Research Council (Vetenskapsrådet, grant 2022-00916)	
Daniel Ferreira - Center for Innovative Medicine (CIMED, grants 20200505 and FoUI-988826)	
Daniel Ferreira - Regional agreement on medical training and clinical research of Stockholm Region (ALF Medicine, grants FoUI-962240 and FoUI-987534)	
Daniel Ferreira - Swedish Brain Foundation (Hjärnfonden FO2023-0261, FO2022-0175, FO2021-0131)	
Daniel Ferreira - Swedish Alzheimer Foundation (Alzheimerfonden AF-968032, AF-980580, AF-994058, AF-1010553)	
Daniel Ferreira - Swedish Dementia Foundation (Demensfonden)	
Daniel Ferreira - the Gamla Tjänarinnor Foundation	
Daniel Ferreira - Gun och Bertil Stohnes Foundation	
Daniel Ferreira - Åke Wiberg Foundation	
Daniel Ferreira - Foundation for Geriatric Diseases at Karolinska Institutet	
Daniel Ferreira - StratNeuro	
Daniel Ferreira - Parkinsonfonden	
Daniel Ferreira - Lindhes	
Daniel Ferreira - Karolinska Institutet	
Daniel Ferreira - Neurofonden	
Daniel Ferreira - contributions from private bequests and academic agreements with industry	
Doug Galasko - NINDS (NIH)	
Neill R Graff-Radford - Eisai	Multicenter grant
Neill R Graff-Radford - Biogen	Multicenter grant
Neill R Graff-Radford - Lilly	Multicenter grant
Neill R Graff-Radford - Cognition therapeutics	Multicenter grant
Samantha K Holden - National Institute on Aging	Paid to institution
Samantha K Holden - Lewy Body Dementia Association	Paid to institution
David Irwin - Alector	Clinical trial research funding to institution
David Irwin - Denali Therapeutics	Clinical trial research funding to institution
David Irwin - Passage Bio	Clinical trial research funding to institution
David Irwin - Prevail	Clinical trial research funding to institution
Alexander Pantelyat - NIA K23 AG059891	Payments to institution
Alexander Pantelyat - NIA R44 AG080861	Payments to institution

		Name all entities with whom you have this relationship or indicate none (add rows as needed)	Specifications/Comments (e.g., if payments were made to you or to your institution)
		Alexander Pantelyat - NINDS U01 NS102035	Payments to institution
		Kathleen L Poston -NIH (U19 AG065156, R01 NS107513, R01 NS115114, P30 AG066515, R01 AG081144, R21 NS132101, U01 DK140939, R01 AG089169)	
		Kathleen L Poston - Michael J Fox Foundation for Parkinson's Research (Grant 020756, 16921, 18411)	
		Kathleen L Poston -The Knight Initiative for Brain Resilience	
		Kathleen L Poston -the Wu Tsai Neuroscience Institute	
		Kathleen L Poston -Lewy Body Dementia Association	
		Kathleen L Poston -Parkinson's Foundation	
		Kathleen Poston - American Parkinson's Disease Association	
		Kathleen L Poston -Sue Berghoff LBD Research Fellowship	
		Sonja W Scholz - National Institute of Neurological Disorders and Stroke	Program #: ZIANS003154
		Holly Shill - Parkinson's Foundation	
		Holly Shill - Barrow Neurological Foundation	
		Holly Shill - NINDS	
		Holly Shill - Michael J Fox Foundation	
		Holly Shill - Intracellular Therapeutics	
		Holly Shill - UCB	
		Juan B Toledo Atucha - NIH	
		Juan B Toledo Atucha - LBDA	
		Juan B Toledo Atucha - Ann and Billy Harrison Foundation	
		Juan B Toledo Atucha - Jerry C. Dearing Family Foundation	
		Alison Yarnall - Dunhill Medical Trust	
		Alison Yarnall - EU IMI	
		Alison Yarnall - NIHR	
		Alison Yarnall - Parkinson's UK	
		Alison Yarnall - Michael J Fox Foundation	
		Alison Yarnall - Weston Brain Institute	
		Alison Yarnall - Intercept Pharmaceuticals	
		Alison Yarnall - Lewy Body Society	
		Alison Yarnall - Cure Parkinson's Trust	
		Alison Yarnall - EPSRC	
		Alison Yarnall - Alzheimer's Society	
		Alison Yarnall - Alzheimer's Research UK	
**3**	Royalties or licenses	☐ **None**	
		Daniel Collerton - Royalty payments for editing Neuroscience of Visual Hallucinations for Blackwell Wiley	Personal
		Fiona Lindop - Parkinson's Disease: A multidisciplinary Guide to Management. Editors	Royalties 2024 & 2025

		Name all entities with whom you have this relationship or indicate none (add rows as needed)	Specifications/Comments (e.g., if payments were made to you or to your institution)
		Fiona LIndiop and Rob Skelly. Published by Elsevier ISBN 9780702082610	
4	Consulting fees	☐ **None**	
		Andrea Bozoki - Cognition Therapeutics	
		Andrea Bozoki - Creative Biopeptides	
		David Bradley - Abbvie consulting fees	
		Paulo Caramelli - Aché	Personal
		Paulo Caramelli - Danone	Personal
		Paulo Caramelli - Knight Therapeutics	Personal
		Daniel Ferreira - BioArtic	
		Doug Galasko - GE Healthcare	Personal
		Samantha K Holden - Contract with Cognition Therapeutics, participation on advisory panel	No payment received yet, will be paid to institution
		Fiona Lindop - Parkinson's UK	Clinical Therapy Lead for Excellence Network – payments to me
		Fiona Lindop - NICE Advice	Payments will be made to me for giving expert advice for a project in 2025
		John O'Brien - Biogen	Acted as Consultant
		John O'Brien - Roche	Acted as Consultant
		John O'Brien - GE Healthcare	Acted as Consultant
		John O'Brien - Okwin	Acted as Consultant
		Alexander Pantelyat - Ono Pharma	Payments to me
		Alexander Pantelyat - Ferrer Internacional	Payments to me
		Alexander Pantelyat - MedRhythms, Inc.	Payments to me
		Alexander Pantelyat - Arvinas, Inc.	Payments to me
		Kathleen L Poston -Novartis	
		Kathleen L Poston -Lilly	
		Kathleen L Poston -BioArctic	
		Kathleen L Poston -Biohaven	
		Kathleen L Poston -Curasen	
		Kathleen L Poston -Neuron23	
		Holly Shill - Boston Scientific	
		Holly Shill - Mitsubishi Tanabe	
		Holly Shill - KeifeRx	
		Juan B Toledo Atucha - GLG Consulting	
		Juan B Toledo Atucha - Spear Bio	
		Juan B Toledo Atucha - Acadia Pharmaceuticals	
		Juan B Toledo Atucha - GE Healthcare	
		Juan B Toledo Atucha - Lundbeck	
		Juan B Toledo Atucha - Newell	
5	Payment or honoraria for lectures, presentations, speakers bureaus,	☐ **None**	
		Laura Bonanni - Eli Lilly	Personal
		Paulo Caramelli - Aché	
		Paulo Caramelli - Biogen	

		Name all entities with whom you have this relationship or indicate none (add rows as needed)	Specifications/Comments (e.g., if payments were made to you or to your institution)
	manuscript writing or educational events	Paulo Caramelli - Danone	
		Paulo Caramelli - Fleury	
		Paulo Caramelli - Lilly	
		Paulo Caramelli - Lundbeck	
		Paulo Caramelli - Novo Nordisk	
		Daniel Collerton - Presentation on Charles Bonnet Syndrome at Association of Hospital Optometrists Annual Meeting 2024	
		Daniel Ferreira - Esteve Pharmaceuticals S.A.	
		Samantha K Holden - Midwestern Medical Directors Association	Honoraria for lecture, paid to self
		Samantha K Holden - Hannover Re	Honoraria for lecture, paid to self
		Clare Johnson - Teach for Royal College of Occupational Therapists (RCOT)	Personal
		Clare Johnson - Teach for Neurology Academy	Personal
		Liam Kennedy - Eisai	Honorarium for presentation on dementia pathophysiology at neurology academy dementia masterclass September 2024
		John O'Brien - GE Healthcare	Received honorarium for lectures
		Holly Shill - Movement Disorder Society	
		Holly Shill - University of Maryland	
		Holly Shill - Parkinson Wellness Recovery	
		Holly Shill - American Academy of Neurology	
		Holly Shill - Parkinson and Movement Disorder Alliance	
		Holly Shill - NeuroCME	
		Juan B Toledo Atucha - Eisai	
		Juan B Toledo Atucha - Fundacio Ace	
		Alison Yarnall - Britannia	Funding and/or honoraria for attending educational events
		Alison Yarnall - UCB	Funding and/or honoraria for attending educational events
		Alison Yarnall - BAP	Funding and/or honoraria for attending educational events
		Alison Yarnall - Abbvie	Funding and/or honoraria for attending educational events
		Alison Yarnall - GSK	Funding and/or honoraria for attending educational events
		Alison Yarnall - Teva-Lundbeck	Funding and/or honoraria for attending educational events
		Alison Yarnall - GE Healthcare	Funding and/or honoraria for attending educational events
		Alison Yarnall - Genus	Funding and/or honoraria for attending educational events
**6**	Payment for expert testimony	☒ **None**	

		Name all entities with whom you have this relationship or indicate none (add rows as needed)	Specifications/Comments (e.g., if payments were made to you or to your institution)
**7**	Support for attending meetings and/or travel	☐ **None**	
		Laura Bonanni - Italian Society of Neurology	
		Laura Bonanni - Italian Society of Neurology - Dementia	
		Laura Bonanni - LIMPE-DISMOV	
		Andrea Bozoki - Kinetix (now PetauriKinect)	
		David Bradley - Abbvie consulting fees	
		Daniel Ferreira - ADPD 2025, ADPD 2024, AAIC 2023	
		Samantha K Holden - Lewy Body Dementia Association	Travel for annual RCOE PI meeting
		Simon Lewis - Movement Disorders Society Faculty Stipend 2022-4	
		Fiona Lindop - Progressive Supranuclear Palsy Association	Reimbursement for travel attending Trustees meeting
		Fiona Lindop - Lewy Body Society - Specialist Advisory Committee	Reimbursement for travel for attending SAC
		Seán O'Dowd - AbbVie Pharma	Support to attend MDS Congress Philadelphia 2023
		Kathleen L Poston -Michael J Fox Foundation for Parkinson's Research	
		Kathleen L Poston -Lewy Body Dementia Association	
		Kathleen L Poston -Parkinson's Foundation	
		Juan B Toledo Atucha - LBDA	
		Alison Yarnall - Britannia	Funding and/or honoraria for attending educational events
		Alison Yarnall - UCB	Funding and/or honoraria for attending educational events
		Alison Yarnall - BAP	Funding and/or honoraria for attending educational events
		Alison Yarnall - Abbvie	Funding and/or honoraria for attending educational events
		Alison Yarnall - GSK	Funding and/or honoraria for attending educational events
		Alison Yarnall - Teva-Lundbeck	Funding and/or honoraria for attending educational events
		Alison Yarnall - GE Healthcare	Funding and/or honoraria for attending educational events
		Alison Yarnall - Genus	Funding and/or honoraria for attending educational events
**8**	Patents planned, issued or pending	☐ **None**	
		Kathleen L Poston -Application No. 17/314,979 filed on May 19, 2021	
		Kathleen L Poston -Application No. 63/377,293 filed on September 27, 2022	
		Sonja W Scholz - A diagnostic biomarker for amyotrophic lateral sclerosis based on plasma protein measurements	U.S. Patent Application No. 63/717,807

		Name all entities with whom you have this relationship or indicate none (add rows as needed)	Specifications/Comments (e.g., if payments were made to you or to your institution)
9	Participation on a Data Safety Monitoring Board or Advisory Board	☐ **None**	
		Andrea Bozoki - AviadoBio Pharma	
		John O'Brien - DSMB	Advisory board
		John O'Brien - TauRx	Advisory board
		John O'Brien - Novo Nordisk	Advisory board
		Alexander Pantelyat - Ono Pharma	Payments to me
		Alexander Pantelyat - MedRhythms, Inc.	Payments to me
		Kathleen L Poston -NCT04497168 (University of Michigan, funded by the NIA/NIH)	
		Alison Yarnall - Data Monitoring Committee, STEPS 2 study	
		Alison Yarnall - Plymouth Hospitals 2023 - present	
10	Leadership or fiduciary role in other board, society, committee or advocacy group, paid or unpaid	☐ **None**	
		Laura Bonanni - Italian society of Neurology	
		Laura Bonanni - Italian society of Neurology-Dementia	
		Laura Bonanni - LIMPE-DISMOV	
		Laura Bonanni - European Academy of Neurology	
		Andrea Bozoki - Dementia Alliance of North Carolina	
		Paul Donaghy - Lewy Body Society Specialist Advisory Committee	Unpaid
		Daniel Ferreira - Director Center Alzheimer Research	
		Samantha Holden - UCNS Behavioral Neurology & Neuropsychiatry Certification Exam Committee	Unpaid
		David Irwin - LBDA Scientific Advisory Board	Unpaid
		David Irwin - AFTD Medical Advisory council	Unpaid
		Fiona Lindop - Progressive Supranuclear Palsy Society Trustee	Unpaid
		Fiona Lindop - Parkinson's UK	Clinical Therapy Lead – personal
		Fiona Lindop - Member of Lewy Body Society Specialist Advisory group until December 2025	Unpaid
		Clare Johnson - Lead on Parkinson's Specialism at RCOT	Unpaid
		Clare Johnson - Therapy rep on BGS MDS SIG	Unpaid
		John O'Brien - UK Alzheimer's Society Research Strategy Council	Chairman
		Seán O'Dowd - Irish Institute of Clinical Neuroscience	Director (unpaid; charitable educational group)
		Alexander Pantelyat - Board of directors, CurePSP	Unpaid
		Holly Shill - Chair, World Parkinson Congress, Local Organizing Committee	
		Juan B Toledo Atucha - Houston Area Parkinson Society	
		Alison Yarnall - Professor of Geriatric Medicine and Honorary Consultant in Older People's Medicine	

		Name all entities with whom you have this relationship or indicate none (add rows as needed)	Specifications/Comments (e.g., if payments were made to you or to your institution)
		Alison Yarnall - Deputy Director Clinical Ageing Research Unit, Newcastle University and Newcastle upon Tyne NHS Foundation Trust	
		Alison Yarnall - DeNDRoN Subspecialty Clinical Research Lead for Parkinson's disease in the North East and Cumbria	
		Alison Yarnall - Newcastle upon Tyne NHS Foundation Trust lead for dementia and delirium	
		Alison Yarnall - Chair of the Digital Outcome Measures subgroup of the EJS ACT-PD Study	
		Alison Yarnall - Member of the International Movement Disorder Society Parkinson's disease-Mild Cognitive Impairment Consortium	
		Alison Yarnall - UK Parkinson's Disease Clinical Studies Group member	
		Alison Yarnall - Deputy Theme lead, Ageing and Multiple Long Term Conditions, Newcastle Heath Tech Research Centre (HRC; 2023 – present)	
		Alison Yarnall - Newcastle Biomedical Research Centre (BRC) Emerging Leader - Dementia and Neurodegeneration (2021 to present)	
		Alison Yarnall - Invited Chair of the national Parkinson's UK Evidence Based Practice committee (January 2019 to 2023)	
**11**	Stock or stock options	☐ **None**	
		Kathleen L Poston -Stock options Amprion	
**12**	Receipt of equipment, materials, drugs, medical writing, gifts or other services	☐ **None**	
		Electrocore provided devices free of charge for my AdVaNSING-PD study	
**13**	Other financial or non-financial interests	☐ **None**	
		Fiona Lindop - My brother-in-law owns Attainability UK who market specialist walking aids for people with Parkinson's (USTEPS and laser canes)	I have no financial or other interest in this
		John O'Brien - Avid/ Lilly	Received academic support for research
		John O'Brien - Merck	Received academic support for research
		John O'Brien - UCB	Received academic support for research
		John O'Brien - Alliance Medical	Received academic support for research
		Sonja W Scholz - Cerevel Therapeutics	Research support

	Name all entities with whom you have this relationship or indicate none (add rows as needed)	Specifications/Comments (e.g., if payments were made to you or to your institution)

Please place an "X" next to the following statement to indicate your agreement:

☒ I certify that I have answered every question and have not altered the wording of any of the questions on this form.
