# Supplementary material for: A common outcome set for trials in dementia with Lewy bodies (DLB COS)
Source: Alzheimers Dement (N Y). 2025 Jul 11;11(3):e70134. doi: 10.1002/trc2.70134 (PMC12254044; doi:10.1002/trc2.70134)
Supplement: Supplementary file 2 — Supporting Information [file TRC2-11-e70134-s004.docx]

**APPENDIX A** Core Outcome Set-STandards for Reporting (COS-STAR) Statement

|  | **SECTION/TOPIC** | **ITEM No.** | **CHECKLIST ITEM** |
| --- | --- | --- | --- |
|  | **TITLE/ABSTRACT** | | |
| ✔ | Title | 1a | Identify in the title that the paper reports the development of a COS |
| ✔ | Abstract | 1b | Provide a structured summary |
|  | **INTRODUCTION** | | |
| ✔ | Background and Objectives | 2a | Describe the background and explain the rationale for developing the COS. |
| ✔ |  | 2b | Describe the specific objectives with reference to developing a COS. |
| ✔ | Scope | 3a | Describe the health condition(s)and population(s) covered by the COS |
| ✔ |  | 3b | Describe the intervention(s) covered by the COS |
| ✔ |  | 3c | Describe the setting(s)in which the COS is to be applied. |
|  | **METHODS** | | |
| ✔ | Protocol/Registry Entry | 4 | Indicate where the COS development protocol can be accessed, if available, and/or the study registration details. |
| ✔ | Participants | 5 | Describe the rationale for stakeholder groups involved in the COS development process, eligibility criteria for participants from each group, and a description of how the individuals involved were identified. |
| ✔ | Information Sources | 6a | Describe the information sources used to identify an initial list of outcomes. |
| ✔ |  | 6b | Describe how outcomes were dropped/combined, with reasons (if applicable). |
| ✔ | Consensus Process | 7 | Describe how the consensus process was undertaken. |
| ✔ | Outcome Scoring | 8 | Describe how outcomes were scored and how scores were summarised. |
| ✔ | Consensus Definition | 9a | Describe the consensus definition. |
| ✔ |  | 9b | Describe the procedure for determining how outcomes were included or excluded from consideration during the consensus process. |
| ✔ | Ethics and Consent | 10 | Provide statement regarding the ethics and consent issues for the study. |
|  | **RESULTS** | | |
| NA | Protocol Deviations | 11 | Describe any changes from the protocol (if applicable), with reasons, and describe what impact these changes have on the results. |
| ✔ | Participants | 12 | Present data on the number and relevant characteristics of the people involved at all stages of COS development. |
| ✔ | Outcomes | 13a | List all outcomes considered at the start of the consensus process. |
| ✔ |  | 13b | Describe any new outcomes introduced and any outcomes dropped, with reasons, during the consensus process. |
| ✔ |  | 14 | List the outcomes in the final COS. |
|  | **DISCUSSION** | | |
| ✔ | Limitations | 15 | Discuss any limitations in the COS development process. |
| ✔ | Conclusions | 16 | Provide an interpretation of the final COS in the context of other evidence, and implications for future research. |
|  | **OTHER INFORMATION** | | |
| ✔ | Funding | 17 | Describe sources of funding /role of funders. |
| ✔ | Conflicts of Interest | 18 | Describe any conflicts of interest within the study team and how these were managed. |
