# Supplementary material for: A common outcome set for trials in dementia with Lewy bodies (DLB COS)
Source: Alzheimers Dement (N Y). 2025 Jul 11;11(3):e70134. doi: 10.1002/trc2.70134 (PMC12254044; doi:10.1002/trc2.70134)
Supplement: Supplementary file 3 — Supporting Information [file TRC2-11-e70134-s005.docx]

**APPENDIX D** PRISMA diagrams

**D1 Quantitative systematic review - PRISMA diagram**

**
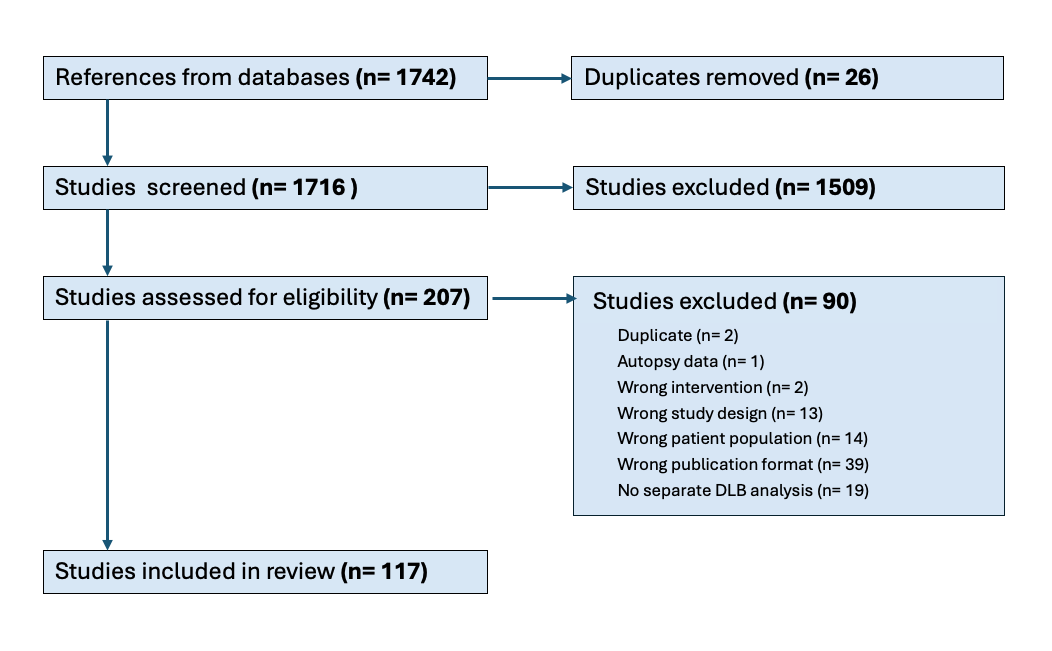
**

**D2 Qualitative systematic review - PRISMA diagram**

**
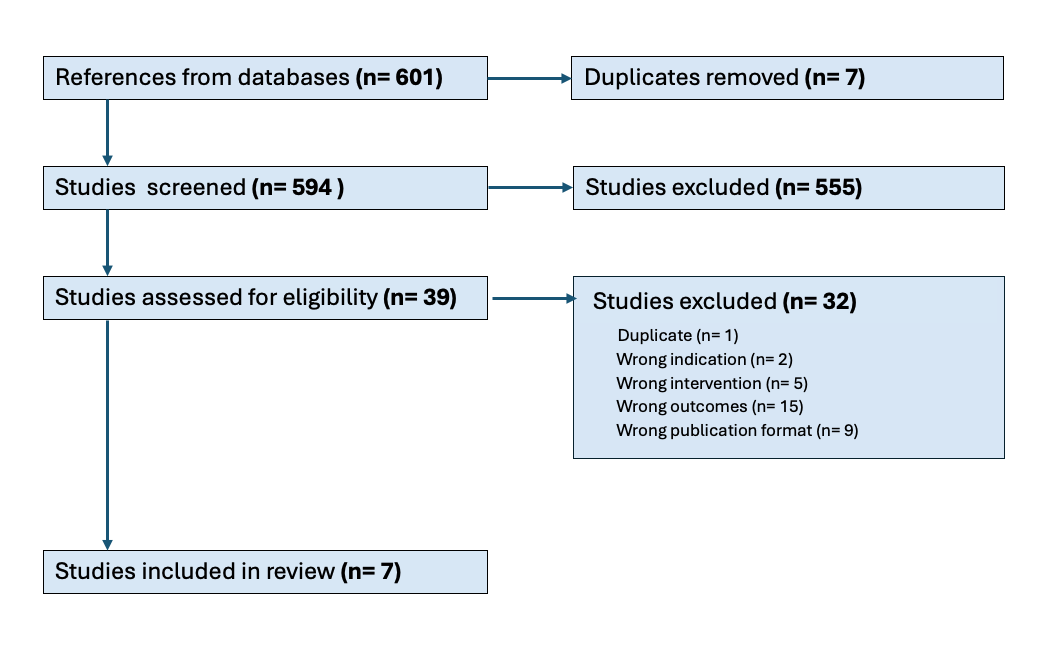
**

**D3 Economic Outcomes systematic review - PRISMA diagram**

**
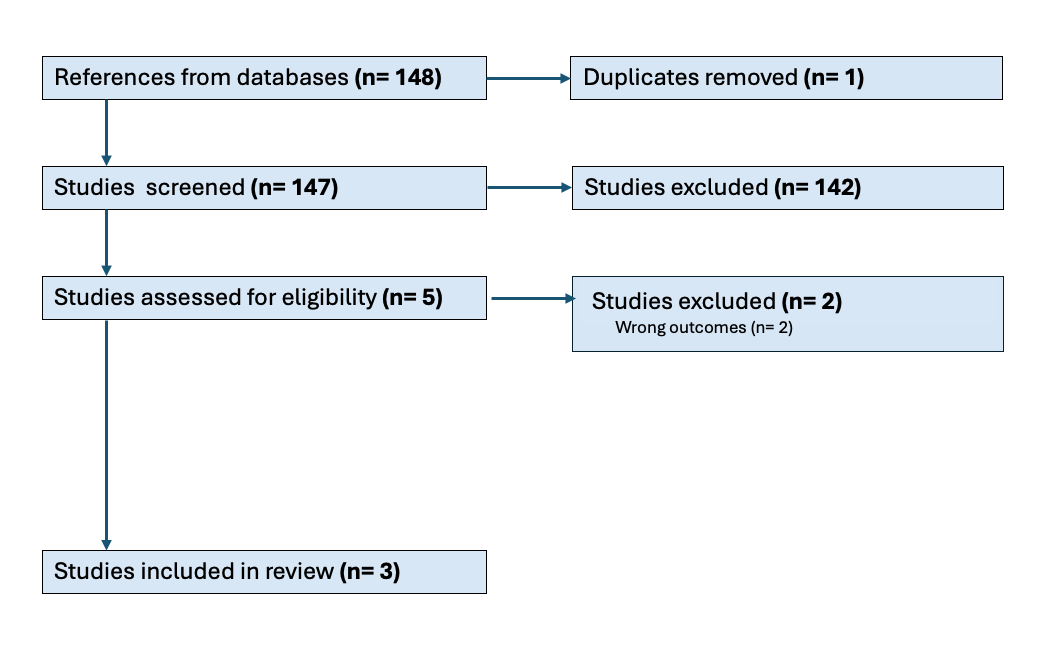
**
