# Supplementary material for: A common outcome set for trials in dementia with Lewy bodies (DLB COS)
Source: Alzheimers Dement (N Y). 2025 Jul 11;11(3):e70134. doi: 10.1002/trc2.70134 (PMC12254044; doi:10.1002/trc2.70134)
Supplement: Supplementary file 4 — Supporting Information [file TRC2-11-e70134-s002.docx]

**APPENDIX B** Search strategies used in systematic reviews

**B1 Quantitative studies systematic review**

### EMBASE

1. ‘Clinical trial’/de
2. ‘Randomized controlled trial’/de
3. Randomization/de
4. ‘Single blind procedure’/de
5. ‘Double blind procedure’/de
6. ‘Crossover procedure’/de
7. Placebo/de
8. ‘Randomi?ed controlled trial*’:ti,ab
9. Rct:ti,ab
10. ‘Random allocation’:ti,ab
11. ‘Randomly allocated’:ti,ab
12. ‘Allocated randomly’:ti,ab
13. (allocated NEAR/2 random):ti,ab
14. ‘Single blind*’:ti,ab
15. ‘Double blind*’:ti,ab
16. ((treble or triple) NEAR/1 (blind*)):ti,ab
17. Placebo*:ti,ab
18. ‘Prospective study'/de
19. #1 OR #2 OR #3 OR #4 OR #5 OR #6 OR #7 OR #8 OR #9 OR #10 OR #11 OR #12 OR #13 OR #14 OR #15 OR #16 OR #17 OR #18
20. ‘Case study’/de
21. ‘Case report’:ti,ab
22. ‘Abstract report’/de or ‘letter’/de OR letter:it OR editorial:it OR 'conference abstract':it OR 'conference review':it
23. #20 OR #21 OR #22
24. #19 not #23
25. 'diffuse Lewy body disease'/exp
26. ('dlb' OR 'dlbd' OR 'lbd' OR 'lewy body dementia*' OR 'lewy body disease*' OR 'dementia with lewy bod*' OR 'diffuse lewy body disease*'):ti,ab
27. #25 OR #26
28. #27 AND #24

### Medline OVID

1. Randomized controlled trials as Topic/
2. Randomized controlled trial/
3. Random allocation/
4. Double blind method/
5. Single blind method/
6. Clinical trial/
7. exp Clinical Trials as Topic/
8. or/1-7
9. (clinic$ adj trial$1).tw.
10. ((singl$ or doubl$ or treb$ or tripl$) adj (blind$3 or mask$3)).tw.
11. Placebos/
12. Placebo$.tw.
13. Randomly allocated.tw.
14. (allocated adj2 random).tw.
15. or/9-14
16. 8 or 15
17. Case report.tw.
18. Letter/
19. Historical article/
20. Review of reported cases.pt.
21. Review, multicase.pt.
22. or/17-21
23. 16 not 22
24. Lewy Body Disease/
25. (dlb OR dlbd OR lbd OR lewy body dementia* OR lewy body disease* OR dementia with lewy bod* OR diffuse lewy body disease*).ti,ab.
26. or/24-25
27. 23 AND 26

### CINAHL

1. TX allocat* random* OR (MH "Quantitative Studies") OR (MH "Placebos") OR TX placebo* OR TX random* allocat* OR (MH "Random Assignment") OR TX randomi* control* trial* OR TX ( (singl* n1 blind*) OR (singl* n1 mask*) ) OR TX ( (doubl* n1 blind*) OR (doubl* n1 mask*) ) OR TX ( (tripl* n1 blind*) OR (tripl* n1 mask*) ) OR TX ( (trebl* n1 blind*) OR (trebl* n1 mask*) ) OR TX clinic* n1 trial* OR PT Clinical trial OR (MH "Clinical Trials+")
2. (MH "Lewy Body Disease")
3. TI (dlbd OR lbd OR “lewy body dementia*” OR “lewy body disease*” OR “dementia with lewy bod*” OR “diffuse lewy body disease*”) OR AB (dlbd OR lbd OR “lewy body dementia*” OR “lewy body disease*” OR “dementia with lewy bod*” OR “diffuse lewy body disease*”)
4. S2 OR S3
5. S1 AND S4

### PsycINFO

1. SU.EXACT("Treatment Effectiveness Evaluation") OR SU.EXACT.EXPLODE("Treatment Outcomes") OR SU.EXACT("Placebo") OR SU.EXACT("Followup Studies") OR placebo* OR random* OR "comparative stud*" OR clinical NEAR/3 trial* OR research NEAR/3 design OR evaluat* NEAR/3 stud* OR prospectiv* NEAR/3 stud* OR (singl* OR doubl* OR trebl* OR tripl*) NEAR/3 (blind* OR mask*)
2. DE "Dementia with Lewy Bodies"
3. TI (dlbd OR lbd OR “lewy body dementia*” OR “lewy body disease*” OR “dementia with lewy bod*” OR “diffuse lewy body disease*”) OR AB (dlbd OR lbd OR “lewy body dementia*” OR “lewy body disease*” OR “dementia with lewy bod*” OR “diffuse lewy body disease*”)
4. S2 OR S3
5. S1 AND S4

### Web of Science

1. TS =(randomised OR randomized OR randomisation OR randomisation OR placebo* OR (random* AND (allocat* OR assign*)) OR (blind* AND (single OR double OR treble OR triple)))
2. TS = (dlbd OR lbd OR “lewy body dementia*” OR “lewy body disease*” OR “dementia with lewy bod*” OR “diffuse lewy body disease*”)
3. #1 AND #2

### Central

1. [mh “Lewy Body Disease”]
2. (dlbd OR lbd OR “lewy body dementia*” OR “lewy body disease*” OR “dementia with lewy bod*” OR “diffuse lewy body disease*”):ti,ab,kw
3. #1 OR #2

**B2 Qualitative studies systematic review**

### EMBASE

1. 'diffuse Lewy body disease'/exp
2. ('dlb' OR 'dlbd' OR 'lbd' OR 'lewy body dementia*' OR 'lewy body disease*' OR 'dementia with lewy bod*' OR 'diffuse lewy body disease*'):ti,ab
3. #1 OR #2
4. 'qualitative research'/de
5. (('semi structured' OR semistructured OR unstructured OR informal OR 'in depth' OR indepth OR 'face to face' OR structured OR guide) NEAR/3 (interview* OR discussion* OR questionnaire*)):ti,ab
6. ('focus group*' OR qualitative OR ethnograph* OR fieldwork OR 'field work' OR 'key informant' OR interview*):ti,ab
7. #4 OR #5 OR #6
8. #3 AND #7

### Medline

1. Lewy Body Disease/
2. (dlb OR dlbd OR lbd OR lewy body dementia* OR lewy body disease* OR dementia with lewy bod* OR diffuse lewy body disease*).ti,ab.
3. or/1-2
4. ((("semi-structured" or semistructured or unstructured or informal or "in-depth" or indepth or "face-to-face" or structured or guide) adj3 (interview* or discussion* or questionnaire*))).ti,ab. or (focus group* or qualitative or ethnograph* or fieldwork or "field work" or "key informant").ti,ab. or interviews as topic/ or focus groups/ or narration/ or qualitative research/
5. 3 AND 4

### CINAHL

1. (MH "Lewy Body Disease")
2. TI (dlbd OR lbd OR “lewy body dementia*” OR “lewy body disease*” OR “dementia with lewy bod*” OR “diffuse lewy body disease*”) OR AB (dlbd OR lbd OR “lewy body dementia*” OR “lewy body disease*” OR “dementia with lewy bod*” OR “diffuse lewy body disease*”)
3. S1 OR S2
4. (MH "cluster sample+") or TX life experiences or TX human science or TX discourse* analysis or TX narrative analysis or TX lived experience* or TX field research or TX field studies or TX field study or TX giorgi* or TX husserl* or TX merleau ponty* or TX van kaam* or TX van manen* or TX spiegelberg* or TX colaizzi* or TX heidegger* or TX participant observ* or TX data saturat* or TX semiotics or TX heuristic or TX hermeneutic* or TX etic or TX emic or TX focus group* or TX purpos* sampl* or TX constant comparison or TX constant comparative or TX grounded research or TX grounded studies or TX grounded study or TX grounded theor* or TX phenomenol* or TX ethnon* or TX qualitative or (MH "ethnological research") or (MH "ethnography") or (MH "phenomenology") or (MH "focus groups") or (MH "discourse analysis") or (MH "theoretical sample") or (MH "field studies") or (MH "constant comparative method") or (MH "thematic analysis") or (MH "content analysis") or (MH "observational methods+") or (MH "purposive sample") or (MH "qualitative validity+") or (MH "grounded theory") or (MH “action research”) or (MH “naturalistic inquiry”) or (MH "ethnonursing research") or (MH "phenomenological research") or (MH "ethnographic research") or (MH "qualitative studies") or (MH "Interviews+") or (MH "Narratives") or (MH "Videorecording+") or (MH "Audiorecording") or (MH "Historical Records")
5. S3 AND S4

### Web of Science

1. TS = (dlbd OR lbd OR “lewy body dementia*” OR “lewy body disease*” OR “dementia with lewy bod*” OR “diffuse lewy body disease*”)
2. TS =(qualitative OR ethnol* OR ethnog* OR ethnonurs* OR emic OR etic OR leininger OR noblit OR "field note*" OR "field record*" OR fieldnote* OR "field stud*" or "participant observ*" OR "participant observation*" OR hermaneutic* OR phenomenolog* OR "lived experience*" OR heidegger* OR husserl* OR "merleau-pont*" OR colaizzi OR giorgi OR ricoeur OR spiegelberg OR "van kaam" OR "van manen" OR "grounded theory" OR "constant compar*" OR "theoretical sampl*" OR glaser AND strauss OR "content analy*" OR "thematic analy*" OR narrative* OR "unstructured categor*" OR "structured categor*" OR "unstructured interview*" OR "semi-structured interview*" OR "maximum variation*" OR snowball OR audio* OR tape* OR video* OR metasynthes* OR "meta-synthes*" OR metasummar* OR "meta-summar*" OR metastud* OR "meta-stud*" OR "meta-ethnograph*" OR metaethnog* OR "meta-narrative*" OR metanarrat* OR " meta-interpretation*" OR metainterpret* OR "qualitative meta-analy*" OR "qualitative metaanaly*" OR "qualitative metanaly*" OR "purposive sampl*" OR "action research" OR "focus group*" or photovoice or "photo voice" or  "mixed method*")
3. #1 AND #2

### PsycINFO

1. DE "Dementia with Lewy Bodies"
2. TI (dlbd OR lbd OR “lewy body dementia*” OR “lewy body disease*” OR “dementia with lewy bod*” OR “diffuse lewy body disease*”) OR AB (dlbd OR lbd OR “lewy body dementia*” OR “lewy body disease*” OR “dementia with lewy bod*” OR “diffuse lewy body disease*”)
3. S1 OR S2
4. DE "Qualitative Methods" OR DE "Focus Group" OR DE "Grounded Theory" OR DE "Interpretative Phenomenological Analysis" OR DE "Narrative Analysis" OR DE "Semi-Structured Interview" OR DE "Thematic Analysis" OR DE "Interviews" OR DE "Group Discussion"
5. TI (("semi-structured" or semistructured or unstructured or informal or "in-depth" or indepth or "face-to-face" or structured or guide or guides) N2 (interview* or discussion* or questionnaire*)) OR AB (("semi-structured" or semistructured or unstructured or informal or "in-depth" or indepth or "face-to-face" or structured or guide or guides) N2 (interview* or discussion* or questionnaire*))
6. TI ("focus group*" or qualitative or ethnograph* or fieldwork or "field work" or "key informant*") OR AB ("focus group*" or qualitative or ethnograph* or fieldwork or "field work" or "key informant*")
7. S4 OR S5 OR S6
8. S3 AND S7

**B3 Economic outcomes systematic review**

### EMBASE

1. 'economic evaluation'/exp
2. 'economic evaluation*':ti,ab
3. 'cost effectiveness analysis'/exp
4. ('cost effectiveness' OR 'cost effectiveness analys?s' OR 'cost effectiveness ratio*' OR 'cost efficiency analys?s'):ti,ab
5. 'cost utility analysis'/exp
6. ('cost utility' OR 'cost utility analys?s'):ti,ab
7. 'cost benefit analysis'/exp
8. ('cost analys?s' OR 'cost benefit' OR 'cost benefit analys?s' OR 'cost benefit ratio*' OR 'cost-benefit analys?s'):ti,ab
9. 'cost consequence analysis'/exp
10. ('cost consequence*' OR 'cost consequence analys?s'):ti,ab
11. 'cost minimization analysis'/exp
12. cost-minimi?ation:ti,ab
13. 'return on investment'/exp
14. 'return on investment*':ti,ab
15. #1 OR #2 OR #3 OR #4 OR #5 OR #6 OR #7 OR #8 OR #9 OR #10 OR #11 OR #12 OR #13 OR #14
16. 'diffuse Lewy body disease'/exp
17. ('dlb' OR 'dlbd' OR 'lbd' OR 'lewy body dementia*' OR 'lewy body disease*' OR 'dementia with lewy bod*' OR 'diffuse lewy body disease*'):ti,ab
18. #16 OR #17
19. #15 AND #18

### Medline

1. economics/
2. value of life/
3. exp "costs and cost analysis"/
4. exp economics, hospital/
5. exp economics, medical/
6. economics, nursing/
7. economics, pharmaceutical/
8. exp "fees and charges"/
9. exp budgets/
10. budget*.ti,ab.
11. cost*.ti.
12. (economic* or pharmaco?economic*).ti.
13. (price* or pricing*).ti,ab.
14. (cost* adj2 (effective* or utilit* or benefit* or minimi* or unit* or estimat* or variable*)).ab.
15. (financ* or fee or fees).ti,ab.
16. (value adj2 (money or monetary)).ti,ab.
17. or/1-16
18. Lewy Body Disease/
19. (dlb OR dlbd OR lbd OR lewy body dementia* OR lewy body disease* OR dementia with lewy bod* OR diffuse lewy body disease*).ti,ab.
20. or/18-19
21. 17 AND 20

### CINAHL

1. (MH "Cost Benefit Analysis")
2. TI (“economic evaluation*”) OR AB (“economic evaluation*”)
3. TI (“Cost consequence analys*” OR “cost-effectiveness”) OR AB (“Cost consequence analys*” OR “cost-effectiveness”)
4. TI (“cost effective” OR “cost benefit*”) OR AB (“cost effective” OR “cost benefit*”)
5. TI (“cost utility”) OR AB (“cost utility”)
6. TI (“cost minimi?ation analysis”) OR AB (“cost minimi?ation analysis”)
7. S1 OR S2 OR S3 OR S4 OR S5 OR S6
8. (MH "Lewy Body Disease")
9. TI (dlbd OR lbd OR “lewy body dementia*” OR “lewy body disease*” OR “dementia with lewy bod*” OR “diffuse lewy body disease*”) OR AB (dlbd OR lbd OR “lewy body dementia*” OR “lewy body disease*” OR “dementia with lewy bod*” OR “diffuse lewy body disease*”)
10. S8 OR S9
11. S7 AND S10

### PSYCINFO

1. DE "Costs and Cost Analysis"
2. TI (“economic evaluation*”) OR AB (“economic evaluation*”)
3. TI (“Cost consequence analys*” OR “cost-effectiveness”) OR AB (“Cost consequence analys*” OR “cost-effectiveness”)
4. TI (“cost effective” OR “cost benefit*”) OR AB (“cost effective” OR “cost benefit*”)
5. TI (“cost utility”) OR AB (“cost utility”)
6. TI (“cost minimi?ation analysis”) OR AB (“cost minimi?ation analysis”)
7. S1 OR S2 OR S3 OR S4 OR S5 OR S6
8. DE "Dementia with Lewy Bodies"
9. TI (dlbd OR lbd OR “lewy body dementia*” OR “lewy body disease*” OR “dementia with lewy bod*” OR “diffuse lewy body disease*”) OR AB (dlbd OR lbd OR “lewy body dementia*” OR “lewy body disease*” OR “dementia with lewy bod*” OR “diffuse lewy body disease*”)
10. S8 OR S9
11. S7 AND S10

### EconLit

1. TI (“economic evaluation*”) OR AB (“economic evaluation*”TI (“Cost consequence analys*” OR “cost-effectiveness”) OR AB (“Cost consequence analys*” OR “cost-effectiveness”)
2. TI (“cost effective” OR “cost benefit*”) OR AB (“cost effective” OR “cost benefit*”)
3. TI (“cost utility”) OR AB (“cost utility”)
4. TI (“cost minimi*ation analysis”) OR AB (“cost minimi*ation analysis”)
5. S1 OR S2 OR S3 OR S4 OR S5
6. TI (dlbd OR lbd OR “lewy body dementia*” OR “lewy body disease*” OR “dementia with lewy bod*” OR “diffuse lewy body disease*”) OR AB (dlbd OR lbd OR “lewy body dementia*” OR “lewy body disease*” OR “dementia with lewy bod*” OR “diffuse lewy body disease*”)
7. S7 OR S8
8. S6 AND S9

### NHS EED

1. MeSH Search: Lewy Body Disease
