# Supplementary material for: A common outcome set for trials in dementia with Lewy bodies (DLB COS)
Source: Alzheimers Dement (N Y). 2025 Jul 11;11(3):e70134. doi: 10.1002/trc2.70134 (PMC12254044; doi:10.1002/trc2.70134)
Supplement: Supplementary file 5 — Supporting Information [file TRC2-11-e70134-s003.docx]

**APPENDIX C** Professional Delphi Members

**Professor Dag Aarsland**

Kings College London, UK

Stavanger University Hospital, Norway

**Dr Panos Alexopoulos**

University of Patras, Greece

**Professor Louise Allan**

University of Exeter, UK

**Dr Jay Amin**

University of Southampton, UK

**Dr Laura Booi**

Leeds Beckett University, UK

**Professor Laura Bonnani**

University G. d'Annunzio of Chieti-Pescara, Italy

**Dr Andrea Bozoki**

University of North Carolina at Chapel Hill, USA

**Professor David Bradley**

Trinity College Dublin, Ireland

**Professor Paulo Caramelli**

Universidade Federal de Minas Gerais, Brazil

**Mr Daniel Collerton**

Newcastle University, UK

**Dr Nilton Custodio**

Peruvian Institute of Neurosciences (IPN) Lince, Peru

**Dr Paul Donaghy**

Newcastle University, UK

**Dr Lidia Engel**

Monash University, Melbourne, Australia

**Dr Daniel Ferreira Padilla**

Karolinska Institutet, Sweden

**Dr Doug Galasko**

University of California San Diego, USA

**Dr Neill R. Graff-Radford**

Mayo Clinic Jacksonville, USA

**Professor Annette Hand**

Northumbria University, UK

**Dr Karen Harrison Dening**

Dementia UK, UK

**Dr Samantha Holden**

University of Colorado, USA

**Dr David Irwin**

University of Pennsylvania, USA

**Ms Clare Johnson**

University Hospitals of Derby and Burton NHS Foundation Trust

**Ms Nisha Joy**

CareBright Community, Ireland

**Dr Liam Kennedy**

Trinity College Dublin, Ireland

**Dr Alison Killen**

Newcastle University, UK

**Professor Simon Lewis**

Macquarie University, Australia

**Mrs Fiona Lindop**

University Hospitals of Derby and Burton NHS Foundation Trust

**Dr Adam McDermott**

Trinity College Dublin, Ireland

**Professor Ian McKeith**

Newcastle University, UK

**Ms Anna Mullen**

St James Hospital Dublin, UK

**Professor John O’Brien**

University of Cambridge, UK

National Institute for Health and Care Excellence, UK

**Dr Seán O’Dowd**

Tallaght University Hospital, Ireland

**Professor Adesola Ogunniyi**

University College Hospital, Ibadan, Nigeria

**Dr Alexander Pantelyat**

Johns Hopkins University, USA

**Florence Pasquier**

Centre Hospitalier Régional Universitaire de Lille, France

**Dr Kathleen Poston**

Stanford University, USA

**Professor Julie Radcliffe**

Flinders University, Adelaide, Australia

**Dr Federico Rodriguez-Porcel**

Medical University of South Carolina, UK

**Dr Sonja W. Scholz**

National Institute of Neurological Disorders and Stroke, National Institutes of Health, USA

**Dr Holly Shill**

Barrow Neurological Institute, USA

**Professor Alan Thomas**

Newcastle University, UK

**Ms Rachel Thompson**

Dementia UK, UK

**Dr Stephen Todd**

Western Health and Social Care Trust, UK

**Dr Jon Toledo**

Nantz National Alzheimer Center, Houston Methodist, USA

**Dr Sridhar Vaitheswaran**

Schizophrenia Research Foundation, Chennai, India

**Professor Mathew Varghese**

National Institute of Mental Health & Neuro Sciences (NIMHANS), Bangalore, India

**Dr Sabina Vatter**

InsideOut Institute, Sydney, Australia

**Dr Alison Yarnall**

Newcastle University, UK

**Dr Kathryn Wyman-Chick**

Struthers Parkinson's Center and Center for Memory and Aging, Minnesota, USA
