# Supplementary material for: A common outcome set for trials in dementia with Lewy bodies (DLB COS)
Source: Alzheimers Dement (N Y). 2025 Jul 11;11(3):e70134. doi: 10.1002/trc2.70134 (PMC12254044; doi:10.1002/trc2.70134)
Supplement: Supplementary file 6 — Supporting Information [file TRC2-11-e70134-s001.docx]

**Appendix E**

Outcomes rated as “*essential for inclusion*” (scored 7-9) by Round 1 respondents

|  | *% of respondents rating symptoms between 7 and 9 (“essential for inclusion”)* | | |
| --- | --- | --- | --- |
|  | *Professional*  *n = 48* | *Lay*  *n = 40* | *All*  *n = 88* |
| **Cognitive ability and fluctuations** |  |  |  |
| Attentional deficits | 66 | 76 | 70 |
| Executive function | 81 | 91 | 85 |
| Fluctuations in cognition | 87 | 79 | 84 |
| Global cognition | 87 | 82 | 85 |
| Language/ communication abilities | 62 | 91 | 74 |
| Memory | 77 | 82 | 79 |
| Social cognition | 53 | 91 | 63 |
| Visuocognition | 77 | 91 | 83 |
|  |  |  |  |
| **Functioning and Quality of Life** |  |  |  |
| Activities of Daily living | 92 | 74 | 85 |
| Care partner burden and distress | 83 | 90 | 86 |
| Care partner mood/ depressive symptoms | 53 | 94 | 69 |
| Care partner quality of life | 62 | 90 | 73 |
| Global Clinical Impression | 83 | 71 | 78 |
| Quality of Life | 85 | 90 | 87 |
|  |  |  |  |
| **Motor and non-motor parkinsonism** |  |  |  |
| Akathisia | 26 | 73 | 46 |
| Falling | 80 | 91 | 85 |
| Gait/walking disturbance | 76 | 85 | 80 |
| Motor parkinsonism | 87 | 88 | 87 |
| On & off states | 35 | 76 | 52 |
| Speech | 50 | 85 | 65 |
| Swallowing | 54 | 94 | 71 |
|  |  |  |  |
| **Psychiatric and sleep-related symptoms** |  |  |  |
| Aggression | 61 | 82 | 70 |
| Agitation | 67 | 88 | 76 |
| Anxiety/ phobias | 70 | 88 | 77 |
| Delusions/ paranoia | 80 | 88 | 84 |
| Depression/ dysphoria | 72 | 91 | 80 |
| Hallucinations | 96 | 94 | 95 |
| Irritability & lability | 44 | 76 | 57 |
| Non-sleep related nighttime behavioral change | 52 | 79 | 63 |
| Sleep | 87 | 94 | 90 |
